# The Role of Mechanotransduction in Contact Inhibition of Locomotion and Proliferation

**DOI:** 10.3390/ijms25042135

**Published:** 2024-02-10

**Authors:** Fumihiko Nakamura

**Affiliations:** School of Pharmaceutical Science and Technology, Tianjin University, 92 Weijin Road, Nankai District, Tianjin 300072, China; fnakamura@tju.edu.cn

**Keywords:** contact inhibition, contact inhibition of locomotion, contact inhibition of proliferation, mechanotransduction, filamin, mechanical force, Yes-associated protein (YAP1), WW domain-containing transcription regulator protein 1 (TAZ), ubiquitin-conjugating enzyme E2 A and B (UBE2A/B), gene expression, proteomics

## Abstract

Contact inhibition (CI) represents a crucial tumor-suppressive mechanism responsible for controlling the unbridled growth of cells, thus preventing the formation of cancerous tissues. CI can be further categorized into two distinct yet interrelated components: CI of locomotion (CIL) and CI of proliferation (CIP). These two components of CI have historically been viewed as separate processes, but emerging research suggests that they may be regulated by both distinct and shared pathways. Specifically, recent studies have indicated that both CIP and CIL utilize mechanotransduction pathways, a process that involves cells sensing and responding to mechanical forces. This review article describes the role of mechanotransduction in CI, shedding light on how mechanical forces regulate CIL and CIP. Emphasis is placed on filamin A (FLNA)-mediated mechanotransduction, elucidating how FLNA senses mechanical forces and translates them into crucial biochemical signals that regulate cell locomotion and proliferation. In addition to FLNA, trans-acting factors (TAFs), which are proteins or regulatory RNAs capable of directly or indirectly binding to specific DNA sequences in distant genes to regulate gene expression, emerge as sensitive players in both the mechanotransduction and signaling pathways of CI. This article presents methods for identifying these TAF proteins and profiling the associated changes in chromatin structure, offering valuable insights into CI and other biological functions mediated by mechanotransduction. Finally, it addresses unanswered research questions in these fields and delineates their possible future directions.

## 1. Introduction

**Figure 1:**
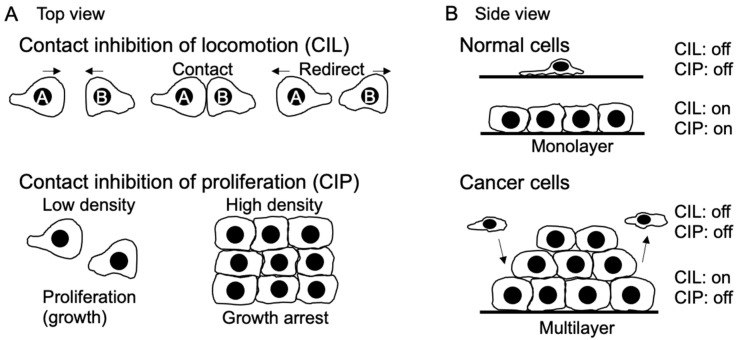
Contact inhibition of locomotion and proliferation in normal and cancer cells. (**A**) Top view: When cells contact each other, the cells change the direction of movement (CIL). At high density, cells stop proliferation (CIP). (**B**) Side view: When normal cells reach the monolayer, the cells stop movement and growth; thereby, both CIL and CIP are on. In cancer cells, metastatic cells migrate (CIL is off) and settle at a target organ (CIL is on). Some cancer cells leave the primary site and move (CIL is off). Cancer cells can grow even at high density (CIP is off).

In 1953, Abercrombie and Heaysman made a significant observation regarding fibroblast cells: when these cells come into contact with each other, their movement velocity decreases, and they change their direction of movement [1,2]. The following year, in 1954, Abercrombie introduced the term “contact inhibition” (CI) to explain why fibroblasts tend to avoid moving over each other’s surfaces and instead form a monolayer [3]. Initially, Abercrombie hypothesized that CI occurred due to the inhibition of cell movement. However, Stoker and Rubin later realized that some cells, such as BHK21 cells, can still grow to form multilayers despite being subject to CI of movement. Therefore, they proposed the term “density-dependent inhibition” to describe the restriction of growth in high-density cells [4]. To distinguish these behaviors, the former is now referred to as CI of locomotion (CIL), and the latter is referred to as CI of proliferation or growth (CIP). Over the past half century, the concept of CI has garnered significant attention, particularly because tumor cells exhibit less sensitivity to CI compared to normal cells [5,6], and CI plays a crucial role in regulating organ size during development and the regeneration process after injury [2] (Figure 1). Despite the attention received, the molecular mechanism of CI has remained elusive until recent times.

The review article of CIL in 2017 remarked that “So far, there is no clear mechanistic connection between CIL and CIP, and they should not be thought of as interrelated processes as some people have suggested” [7]. However, recent evidence has emerged demonstrating the involvement of mechanical forces in both CIL and CIP [8], with some signaling pathways showing overlaps between the two [9]. This suggests the possibility of a mechanistic connection between CIL and CIP, which I will explore and discuss in this article. To illustrate this point, I will use FLNA, an actin cross-linking protein, as an example and delve into how FLNA mediates mechanotransduction, potentially playing significant roles in both CIL and CIP. Mechanotransduction refers to the cellular process through which cells perceive mechanical forces from their environment, as well as forces generated within the cells themselves, and convert them into biochemical and bioelectric signals and subsequent cellular responses, including cell adhesion, shape change, and migration, tissue development, cell proliferation, differentiation, and gene expression [10,11,12]. This intricate process enables cells to sense and adapt to various mechanical cues, such as shear stress, tension, compression, substrate stiffness, and even gravitational forces [13,14,15,16]. Mechanotransduction is attracting attention as a new target for disease treatment and tissue engineering [17,18,19]. Furthermore, I will emphasize the role of TAFs and chromatin structures that are regulated during CI and downstream of mechanotransduction, shedding light on their importance in these processes [20]. Lastly, I will address the remaining questions in these research fields and contemplate potential future directions for further investigation.

## 2. CIL

**Figure 2:**
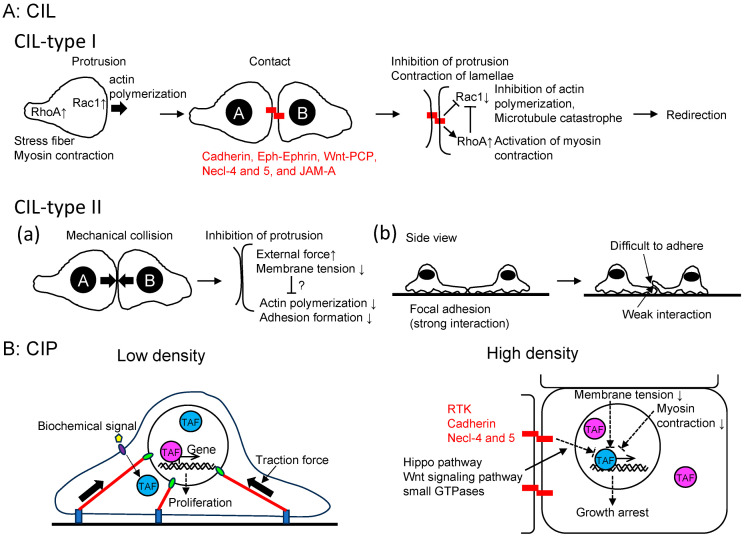
Signaling pathways in CIL and CIP. (**A**) Signaling pathways of CIL. In type I CIL, cell protrusion is inhibited through transmembrane proteins that regulate the balance of RhoA and Rac activities. Wnt-planar cell polarity (PCP) pathway, junctional adhesion molecules (JAMs). In type II CIL, inhibition of protrusion may be regulated by (**a**) force generated by collision and alteration of membrane tension, and/or (**b**) loss of adhesion to a colliding cell. (**B**) Signaling pathways of CIP. At low cell density, TAFs (e.g., transcriptional coactivator YAP1, with the transcription factor represented in magenta and the repressor in cyan) bound to chromatin either induce or suppress gene expression for proliferation, respectively. Both biochemical signals and mechanical forces control the activation of genes. At high density, transmembrane proteins, membrane tension, and the relaxation of myosin contraction promote the translocation and dissociation/association of TAFs to chromatin, leading to growth arrest. Some TAFs may bind to chromatin only in high-density cells, although such TAFs have not yet been identified. Receptor tyrosine kinase (RTK).

The loss of CIL enables malignant cells to invade normal cell clusters within tissues. A comprehensive review article on CIL was published by Stramer and Mayor in 2017 [7], prompting me to focus on the advancements in this field over the past seven years. In their review article, CIL was categorized into at least two types (Figure 2A). Type I arises from the suppression of cell protrusive activity at the contact site, causing leading-edge contraction and migration away from the point of collision. For example, zebrafish endodermal cells undergo homotypic Type I CIL [21]. Conversely, Type II occurs when a cell struggles to move over another cell due to weaker adhesion with the neighboring cell compared to the substratum [22]. Regulation of type I CIL involves cell surface transmembrane proteins and downstream signaling molecules like small GTPases [22]. Meanwhile, type II CIL appears to be primarily influenced by membrane tension and external forces, which also induce cytoskeleton remodeling. Since the publication of the review article, several additional players, such as cadherin, Eph-Ephrin, Wnt, protein tyrosine kinase 7 (PTK7), and junctional adhesion molecule-A (JAM-A), have been discovered to contribute to type I CIL [23,24,25,26,27,28]. For instance, the binding of Eph receptors to their ephrin ligands at cell–cell contact sites leads to the tyrosine phosphorylation of ephrinB2 and the disruption of the ephrinB2/Dsh/TBC1d24 complex. Consequently, this complex disruption results in an increase in E-cadherin levels at the plasma membrane, leading to the loss of CIL and subsequent regulation of neural crest cell migration (collective migration) [23]. The deletion of extracellular domains of Cadherin3 in Xenopus mesendodermal cells disrupts CIL by reducing Rac1 activity, implying the involvement of adhesion-independent signaling by Cadherin3 [25]. However, the specific molecular mechanism through which non-junctional Cadherin3 regulates Rac1 activity remains unknown. In MCF7 mammary epithelial cells derived from human breast adenocarcinoma, when the leader cells of migrating sheets come into head-on contact, it induces changes in retrograde cortical actin flow within lamellipodia. Additionally, antiparallel cortical flows are observed at the lamellipodia of colliding cells, exerting tension on the α-catenin actin-binding domain, leading to increased adhesion growth. Subsequently, mechanical loading results in the downregulation of cortical flow, effectively immobilizing lamellipodia and inhibiting cell locomotion [28]. PTK7 is an evolutionary conserved transmembrane protein with an intracellular kinase homology domain that lacks catalytic activity. It also functions as a Wnt co-receptor and plays a crucial role in neural crest migration [26]. PTK7 is found at cell–cell contact sites of migrating Xenopus neural crest cells and is essential for CIL.

On the other hand, JAM-A, localized at membrane protrusions in migrating cells, associates with αvβ5 integrin through the tetraspanins CD9 and/or CD81 [27]. In this state, Rac1 remains active, promoting cell protrusion and migration. However, upon cell contact with colliding cells, JAM-A clusters and inhibits Src and Rac1 activities, effectively suppressing cell migration. It is worth noting that the outcome of such collisions appears to be predictable. For instance, cells with a smaller contact angle (indicating a broader spread of lamellipodia) relative to the surface or those with higher speeds are more likely to maintain their direction after a collision [29,30].

CIL plays a role in regulating the assembly and disassembly of microtubules (MTs) through coupled Rho GTPase signaling, thus influencing actin remodeling, adhesion, and myosin-mediated contraction as well [31]. Furthermore, there is crosstalk between MTs and the actin cytoskeleton through their shared binding partners [32,33]. However, the specific involvement of these molecules in CIL remains relatively understudied.

Type II CIL’s mechanism has received less attention in research. Nonetheless, it has been observed that even weak forces can stall protrusion at the leading edge of the lamellipodium [34], and protrusion events are restricted by membrane tension [35].

### 2.1. CIL in Different Dimensional Environments

CIL, originally observed in two-dimensional tissue culture cells, plays a vital role in three-dimensional settings as well, influencing animal development and cancer invasion. In the collective migration of mesenchymal cells like neural crest cells, CIL works by inhibiting lamellipodial protrusions at cell–cell contacts and facilitating polarization at the leading edge [36]. However, different cells employ distinct modes of collective migration, either filopodia-based or lamellipodia-based [37]. Interactions between different cell types can induce specific forms of CIL, known as heterotypic CIL, which leads to cell sorting between epithelial and mesenchymal cell populations [24]. For instance, when migrating epithelial cells (HaCaT) come into contact with migrating fibroblasts (NIH3T3), both cells halt their forward motion. In contrast, fibrosarcoma cells (HT1080) experience repulsion after colliding with epithelial cells (HaCaT). This repulsion is prevented when EphB2 or ERK is knocked down in fibrosarcoma cells.

Cells on fibers that mimic the in vivo extracellular matrix (ECM) exhibit distinct behaviors compared to cells on 2D surfaces [38]. When approaching cells are attached to a single fiber, they do not repolarize upon contact but rather migrate past each other, continuing along their respective directions. However, when two parallel cells attached to two fibers with a spacing of approximately 10 µm come into contact, one cell will repolarize, change its migration direction, and move together with the other. Additionally, if a trailing cell moves faster and contacts the leading cells, the leading cells will speed up in response [38]. As a result, conventional CIL, which occurs when the heads of migrating cells come into contact on 2D surfaces, does not occur on fibers. Instead, contacts between different regions of cells elicit varied responses. For example, when a trailing cell contacts the tail of a neighboring cell, the trailing cell migrates towards the tail. This behavior is known as “contact following of locomotion” (CFL) and is implicated in collective migration regulated by the Wnt signaling pathway [39]. Inhibition of the Wnt pathway disrupts both CFL and the collective migration of epithelial cells. Consequently, both CIL and CFL play vital roles in controlling collective cell migration within a multicellular environment [40].

### 2.2. Cell Rigidity Transition between Jamming and Unjamming in Collective Cell Migration

During wound healing, embryogenesis, and tumor invasion, only a portion of epithelial cells engage in directed movement, while others remain in a jammed or solid-like state [41]. In contrast, the majority of mesenchymal cells are actively migratory and do not experience this jammed state. The transition from a jammed to a more fluid or unjammed state occurs in various physiological and pathological contexts where cell mobility and adaptability are essential. This transition, which is different from the epithelial-to-mesenchymal transition, often leads to changes in cell behavior, such as CIL, increased mobility, and the ability to move collectively. For instance, in densely populated cellular environments, jamming can result from physical crowding. Unjamming might happen as cells rearrange or exert forces to open up spaces between neighboring cells, thus facilitating more fluid movement. The molecular mechanisms behind this transition are starting to be understood. For example, RAB5A, which is elevated by various factors such as growth factors, stress, and oncogenic factors, enhances non-clathrin-mediated endocytosis of the epidermal growth factor receptor (EGFR), leading to its accumulation in endosomal vesicles. These vesicles then act as signaling hubs for sustained and heightened ERK1/2 activation. This activation is enough to cause hyper-phosphorylation of WAVE2, which plays a role in controlling actin polymerization. This contributes to the dynamics of cell protrusions and leads to the unjammed state [42,43].

Unjamming is also linked to mechanotransduction, as the ability of cells to unjam is influenced by mechanical signals. For example, the formation of cortical branched actin by Arp2/3 complexes containing ARPC1B is regulated by the RAC/WAVE/ARPIN pathway, which is stimulated by growth factors and mechanotransduction cues from cell adhesions. These ARPC1B-containing complexes are specifically targeted by coronin-1B, which plays a key role in controlling the cell cycle progression in mammary epithelial cells. The function of the Arp2/3 complex is essential for maintaining migration persistence, which refers to the ability to keep a consistent direction over time. Interestingly, cells that exhibit greater persistence in migration are also those that undergo faster cell cycling. On the other hand, complexes containing ARPC1A are vital for the formation of endosomal branched actin, yet they do not influence the progression of the cell cycle [44].

In multicellular organisms, cells engage in interactions with tissue structures such as the ECM and neighboring cells. The molecular mechanisms underlying the transition between jamming and unjamming in this intricate environment require further in-depth research [45].

## 3. CIP

The Hippo pathway plays a vital role in controlling cell proliferation by phosphorylating the transcriptional co-activator YAP1/TAZ [46], subject to regulation by multiple upstream factors [47], although YAP and TAZ exhibit different translocation dynamics in some cases [48]. Biochemical signals and mechanical forces both control the Hippo pathway [49] (Figure 2B). At low cell density, there is an increased production of amphiregulin, an EGFR ligand. This subsequently reduces Hippo pathway signaling, leading to the nuclear translocation of YAP1/TAZ. This process establishes a positive autocrine or paracrine feedback loop that encourages proliferation [50]. On the contrary, at high cell density, confluent cells are expected to exhibit more paracrine and ECM signaling due to the increased secretion of paracrine factors and deposition of ECM. However, when low-density cells are cultured in tissue culture supernatant from high-density cell culture, they do not cease proliferation. Moreover, some cancer cells continue to grow even at high density, suggesting that the paracrine factors from high-density cells alone are not sufficient to regulate CIP. Instead, biochemical signaling for CIP is mediated by adhesion receptors such as E-cadherin. While E-cadherin regulates CIL through small GTPases and cytoskeletons, CIP is controlled through multiple pathways, including growth factor receptors, merlin, the Hippo pathway, the Wnt signaling pathway, and small GTPases [51,52]. High cell density inhibits growth factor receptor signaling, such as EGFR and insulin-like growth factor 1 receptor, in an E-cadherin-dependent manner. In other words, CIP reduces sensitivity to growth factors [53]. Overexpression of E-cadherin or an increase in cell density leads to the suppression of cell growth by raising the threshold level of the growth factor required for proliferation. Conversely, in E-cadherin knockdown cells, CIP is eliminated, suggesting that growth factor-induced cell proliferation depends on E-cadherin rather than an increase in cell density [51]. Interestingly, cleavage of the cytoplasmic domains of E-cadherin and N-cadherin by γ-secretase can abolish CIP and induce anchorage-independent growth [54]. It is worth noting that while the cytoplasmic domains of cadherins induce YAP1/TAZ translocation to the nucleus, the expression of downstream genes like TAZ and connective tissue growth factor (CTGF) remains unaffected. This suggests that YAP1 nuclear localization alone is insufficient as an indicator of YAP1 activity, and additional factors are required to activate YAP1/TAZ. Supporting this observation, Yap1 S112A mice (corresponding to human YAP1 S127) are surprisingly normal despite the nuclear localization of the mutant YAP1, possibly due to homeostatic mechanisms that maintain physiological levels of YAP1 activity [55]. In addition, recent research suggests that the dynamic entry and exit of YAP1 define its activity. More specifically, the majority of Yorkie (the Drosophila ortholog of YAP1) dynamically fluctuates between the cytoplasm and nucleus, and a cycle of fast exit of nuclear YAP1 to the cytoplasm followed by fast re-entry to the nucleus (“localization-resets”) activates YAP1 target genes [56]. Furthermore, extracellular signal-regulated kinase 5 (ERK5)/mitogen-activated protein kinase 7 (MAPK7) are essential for YAP1/TEAD interaction and YAP1 recruitment on DNA in liver cells [57]. Additionally, Necl-4 and 5 regulate CIP through ERK1/2 (MAPK3/MAPK1) [58] (Figure 2). Recently, JAM-A has been found to interact with merlin and large tumor suppressor kinases (LATS1/2), thereby activating the Hippo signaling pathway to suppress proliferation [59].

Mechanical forces can also influence the translocation of YAP1 in a Hippo-independent manner [60,61]. Even when the phosphorylation of YAP1 is inhibited by deleting LATS1/2, YAP1 remains in the cytosol of cells on a soft substrate [60]. This suggests that dephosphorylation of YAP1 alone is not sufficient for its nuclear entry, and a mechanical cue is necessary. The force-induced stretching of the nuclear pore complex (NPC), mediated by the linker of the nucleoskeleton and cytoskeleton (LINC) complex and actin cytoskeleton, is responsible for promoting the nuclear entry of YAP1 [62]. On soft substrates, alterations in substrate stiffness specifically impact the nuclear entry of YAP1, while leaving its export unaffected. Consequently, the nuclear import of YAP1 is lower than its export on soft substrates. Conversely, on stiff substrates, the actin cytoskeleton stretches and curves the NPC, exposing its cytoplasmic side, which enhances YAP1 import. The interaction of YAP1 with importin 7 is crucial for the nuclear entry of YAP1, but this interaction is abolished by the phosphorylation of YAP1 on serine 127 (or 112 in mice) by LATS1/2, induced by inhibition of myosin and high cell density [63]. These findings suggest that the mechanical cue remains crucial even for the Hippo-dependent nuclear entry of YAP1. Even in the presence of the S127A mutant YAP1, which is not affected by LATS-dependent phosphorylation, the nuclear entry of YAP1 is still sensitive to substrate rigidity and mechanical signals [62].

Recent research has revealed a more intricate mechanism governing the nuclear entry of YAP1. YAP1 interacts with Enigma and Enigma-like proteins (PDLIM7 and PDLIM5, respectively) through their C-terminal PDZ-binding motif, which is vital for YAP1’s full nuclear localization and activity. Silencing the expression of PDLIM5/7 leads to a reduction in YAP1’s nuclear entry and transcriptional activity, indicating that the opening of the NPC alone is not enough for YAP1’s nuclear entry [64]. Enigma PDLIM7 predominantly localizes to the cytoplasm in densely populated cells. However, in low-density cells, it translocates to F-actin stress fibers, focal adhesions, and F-actin fibers at adherens junctions through alpha-actinin. This localization promotes the tyrosine phosphorylation of YAP1 by Src family kinases, including Yes, in the integrin-Src signaling complex, leading to the activation of YAP1. Phosphorylation on tyrosine 357 sets YAP1 apart from Hippo-dependent phosphorylation on serine 127, as it triggers YAP1 activation and translocation to the nucleus, where it forms a transcriptionally active complex [65]. Within the integrin-Src-mediated mechanotransduction, ANKHD1 and ANKRD17, members of the mask family proteins, play a vital role in facilitating YAP1’s nuclear entry [66]. These proteins possess two ankyrin repeat domains that bind to YAP1 and also contain a conserved nuclear localization sequence and nuclear export sequence, which together regulate YAP1’s nuclear import and stability. In Drosophila epithelia, physiological mechanical strain at the apical membrane has been shown to decrease Hippo kinase dimerization, leading to downregulation of Hippo signaling and activation of Yki (YAP1) [67]. However, it remains to be investigated whether this mechanism can be applied to animal cells.

Inhibiting exportin1 prevents the translocation of YAP1 to the cytoplasm, irrespective of substrate stiffness [60,62]. This suggests that active transport is essential for the nuclear exit of YAP1. Interestingly, stretching the NPC does not appear to be necessary for the export, as inhibition of myosin and high cell density do not hinder nuclear exit. Thus, YAP1 can exit the nucleus even when the NPC is not stretched. In cells on a soft substrate or at high density, YAP1 is scarcely detected in the nucleus, indicating that the exported YAP1 is sequestered in the cytoplasm. This sequestering process may involve inhibition of YAP1’s entry into the nucleus and its binding to 14-3-3 proteins, which occurs after YAP1’s phosphorylation in a Hippo-dependent or -independent manner [68]. Moreover, AMOT plays a role in sequestering YAP1 in the cytoplasm. AMOT directly binds to F-actin, but when F-actin depolymerizes, AMOT is released and binds to YAP1 [69]. Such depolymerization occurs in cells treated with latrunculin A at high density. Phosphorylation of AMOT by LATS kinase promotes its dissociation from F-actin and inhibits cell proliferation [70].

Significantly, these recent findings emphasize the existence of overlapping as well as independent pathways between CIL and CIP (Figure 2). Nevertheless, it is noteworthy that the stretching of dense cells alone can lead to YAP1’s nuclear localization [61], indicating that cell–cell contact alone is inadequate to retain YAP1 in the cytoplasm. In addition, tissue confinement (i.e., cell volume) also determines YAP1 localization and cell growth [71].

### 3.1. The Role of Mechanotransduction in CIP for the Regulation of Gene Expression

External or internal forces, generated during CIP, regulate gene expression via mechanotransduction. External forces can be sensed by mechanosensitive channels like Piezo and TRP families [72] and other transmembrane proteins, including integrins, glycocalyx, and anthrax toxin protein receptor 1 [73,74]. These forces induce conformational changes in the channels, opening them to allow the influx of ions that stimulate downstream signaling events [75]. For instance, the activation of Piezo1 induces an influx of calcium ions, subsequently activating calcium-dependent myosin II [76]. The mechanical force that triggers Piezo1 activation also originates from activated myosin II [77], indicating the presence of a feedback loop that sustains Piezo1 activation. Interestingly, not only activation but also expression of Piezo1 is mechanosensitive, while expression of Piezo2 appears to be less mechanosensitive [78] and is distinct from Piezo1 for some functions [79]. Additionally, Piezo1 activates YAP1, and its knockout leads to increased phosphorylated YAP1 [79].

Calcium ions can also activate other signaling pathways to regulate gene expression [80]. For example, the binding of calcium ions to DREAM disrupts its tetramer formation, leading to the derepression of gene expression [81]. Furthermore, calcium ions might influence epigenetic modifications like DNA methylation and alternative splicing patterns, thereby modulating gene expression [80]. These findings indicate that cell–cell contact-induced relaxation of the plasma membrane could inactivate mechanosensitive channels, halting growth by altering gene expression. Glycocalyx and integrins also participate in mechanotransduction to regulate gene expression. For instance, fluid shear stress induces remodeling of the endothelial glycocalyx, activating gene expression [82]. Similarly, tensile force applied to integrins at the focal adhesion site promotes the activation of focal adhesion kinase and Src kinase, connecting to the LINC complex through the cytoskeleton [83]. This connection stretches the nuclear pore complex, facilitating the nuclear translocation of YAP1 to induce gene expression [62,84].

Mechanosensing can occur not only at the plasma membrane but also at the cellular organelles. For example, the mechanical properties of the nucleus and mitochondria can be altered by internal or external forces [85]. The internal mechanical forces can be generated by myosin contraction and polymerization of the cytoskeleton. Inhibition of myosin II by a specific inhibitor, blebbistatin, or by inhibition of upstream Rho kinase by Y27632 prevents nuclear localization of YAP1 and UBE2A/B [60,86,87]. Treatment that disrupts actin polymerization also opposes nuclear localization of YAP1 and UBE2A/B, presumably because myosin cannot generate force without filamentous actin (F-actin) and/or pushing force generated by polymerization is reduced. Surprisingly, the depletion of F-actin-capping/severing proteins such as cofilin, capZ, or gelsolin is individually sufficient to rescue YAP1 nuclear localization and YAP1-dependent gene expression in high-density cells and cells on a soft substrate [61]. However, it is worth noting that transgenic gelsolin-null mice appear to have normal embryonic development and longevity [88].

**Figure 3:**
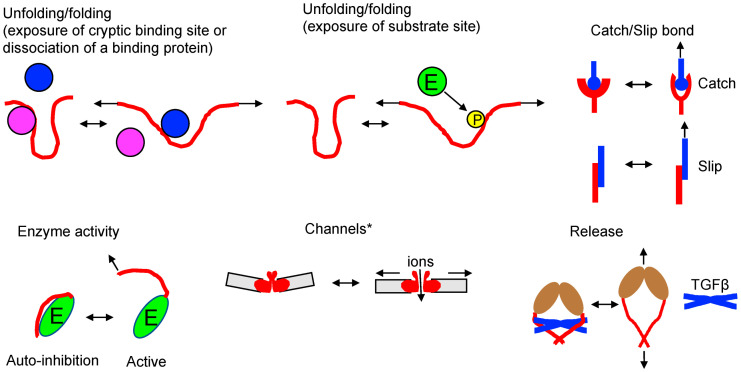
Molecules that change conformation by mechanical forces.

The typical pushing forces generated by a growing individual F-actin and microtubule are in the range of 0.76 ± 0.22 pN and 3–4 pN, respectively [89,90,91]. In comparison, single myosin, kinesin, and dynein motors can generate forces of approximately 3–4 pN, 5–6 pN, and ~7 pN forces, respectively, though these values may vary depending on the methods used [92,93,94]. This range of forces appears to be sufficient to induce conformational changes in mechanosensitive protein molecules (Figure 3) [95].

Unfolding-folding—Force-induced conformational changes can expose a cryptic binding site or dissociate a binding protein. During the conformational changes of the protein (red), some proteins (magenta) may dissociate, while others (blue) may bind to the exposed binding site. Additionally, unfolding of the protein (red in the upper middle) may also expose the substrate site (E: enzyme, P: phosphate group). Examples include talin [96,97,98], filamin [99,100], vinculin [101], alpha-catenin [102,103], alpha-actinin 1 [104], lamin A/C [105], von Willebrand factor [106,107], fibronectin [108,109,110]) or substrate site (phosphorylation of p130Cas [111,112], FAK [113], PDZ proteins [114].

Catch-slip bond—Catch bonds resist dissociation when subjected to mechanical force, leading to longer lifetimes with increasing force (top of the upper right). Several models have been proposed to explain catch bonds in biological systems, and the exact molecular mechanisms underlying catch bonds can vary between different systems and molecules. On the other hand, slip bonds weaken or have shorter lifetimes when exposed to mechanical force (bottom of the upper right). Examples include integrin [115,116], L-selectin [117], P-selectin glycoprotein ligand 1 (PSGL-1)/P-selectin [118], TCR/pMHC [119,120,121], LFA-1/ICAM-1 [122], cadherin-catenin complex/F-actin [123], and DNAM-1/CD155 [124]. Catch bond engineering can be employed to tune high-sensitivity TCRs for T cell therapy [125].

Enzyme activity—Some enzymes have specific structural domains or regions that are sensitive to mechanical force. Application of mechanical force to an enzyme can lead to conformational changes in certain regions. Such conformational changes may the expose enzyme’s active site, promote the binding of substrates to the enzyme, increase the rate of enzymatic reactions, induce the activation of allosteric sites, and trigger downstream signaling pathways or enzymatic activities. For example, mechanical force exposes the catalytic site of titin kinase by rearranging the autoinhibitory tail [126]. Forces generated via the actin cytoskeleton in focal adhesions release the kinase domain of focal adhesion kinase (FAK) from the FERM domain [113].

Channels—Mechanosensitive channels, also known as stretch-activated ion channels, are proteins found in the membranes of various cells. They play a critical role in sensing and responding to mechanical forces, such as pressure, tension, or stretching of the cell membrane. The molecular mechanism of mechanosensitive channels involves their structure and conformational changes in response to mechanical force. * Different mechanosensitive channels use different mechanisms to open channels [72,127,128]. Examples include piezo1/2 [129], transient receptor potential channels (TRPs: TRPM, TRPV, TRPC) [130,131,132], TRAAK [133], TREK [134,135], BK channels [136], OSCA/TMEM63 [137], and DEG/ENaC [138].

Release—Mechanical force transmitted through F-actin can influence the activation of integrin αVβ6 and its interaction with pro-TGF-β1, a pivotal step in the activation and signaling of TGF-β1. In its dormant state, integrin αVβ6 exhibits minimal affinity for its ligands, including pro-TGF-β1, and adopts a bent or closed conformation, with the extracellular domains of the αV and β6 subunits closely associated. The application of mechanical force, such as tensile force or shear stress, to the cell or tissue can induce conformational changes in integrin αVβ6. This process exposes cryptic binding sites and the RGD (Arg-Gly-Asp) motif within pro-TGF-β1. The binding of pro-TGF-β1 to activated integrin αVβ6 under mechanical force conditions facilitates the localized release and activation of TGF-β1. This activation process typically involves the cleavage of the pro-peptide from pro-TGF-β1, converting it into its active form [139,140].

For example, the cryptic vinculin-binding site of talin can be exposed by 2–12 pN of stretching force [96] and the filamin mechanosensing domain can be exposed with 2–5 pN force [141]. Unfolding of α-catenin can occur at 5–15 pN force to promote vinculin binding [103]. Membrane-embedded Piezo1 channel can open in the range of 6–50 pN force applied to the supporting membrane [129]. In addition, it was observed that the average force exerted by a single vinculin molecule in stationary focal adhesion is approximately ~2.5 pN [101]. While a single motor protein may not generate sufficient force, the presence of multiple motors could result in much stronger forces. Since actin–actin bond breaking force and the minimum force required to rupture a microtubule are estimated to be around ~600 pN and ~500 pN, respectively [142,143], even with the collective force from multiple motor proteins, these filaments do not break. These findings strongly suggest that mechanotransduction can indeed be regulated by physiological forces within cells [144].

When cells exert pressure on each other, stress stiffening and stress softening of actin filaments in the leading edge may arise due to the resistance of actin filaments and their buckling against compression [145]. Although the potential impact of these changes on mechanotransduction has been hypothesized, it has not been demonstrated yet.

**Figure 4:**
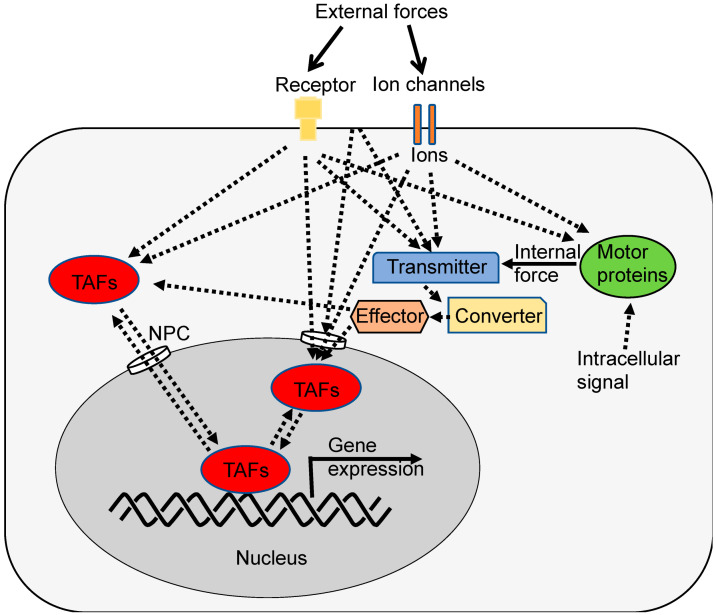
Mechanotransduction in animal cells.

It seems that the mechanical stretching of NPC is crucial, at least for the nuclear entry of YAP1 [62]. Nevertheless, it is possible that signaling molecules downstream of mechanotransduction or cell–cell contact could facilitate the nuclear entry of other TAFs without altering NPC (Figure 4). For instance, small molecules with a molecular weight of less than 20 kDa might be less sensitive to force-induced NPC opening and could freely enter the nucleus unless import and export are actively regulated [62]. Additionally, other regulatory mechanisms, such as post-translational modifications or ion binding, might also control nuclear entry. Furthermore, it is feasible that signals downstream of mechanotransduction, or CIP, directly stimulate TAFs within the nucleus (Figure 4). For example, calcium ions might directly or indirectly activate nuclear TAFs [80]. However, as of now, such mechanisms have not been demonstrated.

Hypothesis: How mechanical forces are converted to biological signals. Cells can experience mechanical forces from various sources. External forces may include physical cues from the ECM, neighboring cells, or fluid shear stress in blood vessels. Internal forces can originate from processes within the cell, such as cytoskeletal tension or organelle movements. Mechanosensitive proteins such as integrins, piezos, and talin can detect changes in tension, compression, or shear within or around the cell. Once detected, the mechanical signals are transmitted to the next components of the mechanotransduction pathway. Converter (e.g., FLNA), effector (e.g., FilGAP), or regulator (e.g., NPC and Channel) are elements within the cell that act as intermediaries in the mechanotransduction process. They receive the mechanical signals and convert them into biochemical or biophysical responses. TAFs are molecules capable of directly or indirectly binding to specific DNA sequences in distant genes to regulate gene expression within the cell that can be affected by mechanical signals. TAFs include not only activator and repressor transcription factor proteins but also long noncoding RNAs (lncRNAs) such as Neat1 [146]. In this context, YAP1 and TAZ are examples of TAFs. These factors are known to regulate gene expression and cellular behaviors in response to mechanical cues. The localization of YAP1 and TAZ within the cell can be controlled by mechanical forces, which can determine their activity. Mechanical forces can also affect ion channels and transporters. Ion influx, or the movement of ions (such as calcium or potassium) into the cell, can be regulated by mechanical signals. This ion flux can, in turn, influence various cellular processes, including cell signaling and gene expression. FLNA acts as a sensor, transmitter, and converter [99]. These converted signals eventually regulate cell growth, differentiation, cell adhesion, shape change, migration, metabolism, etc.

The stretching of NPC facilitates the passive transport of large molecules, but this effect diminishes as the size of the molecule increases, which may account for the differential extent of nuclear entry for UBE2A/B (152aa) and YAP1 (504aa) [62,87]. The NPC stretching model proposes that not only YAP1 but also other proteins could potentially move between the nucleus and cytoplasm upon mechanical stimulation. Up to now, a total of 41 nucleocytoplasmic shuttling molecules sensitive to mechanical forces and/or cell density have been identified (Table S1) [147]. Surprisingly, even relatively large molecules (e.g., SREBP1: 1147aa) can shuttle, indicating that active transport assists in the translocation of large molecules. According to this model, even more nucleocytoplasmic shuttling molecules could be involved in CI and mechanotransduction because there are many small proteins involved in gene regulation. However, the identification of such molecules faces significant challenges, primarily due to the lack of appropriate methodologies. Generally, TAFs such as transcription factors (TFs) and their binding proteins are expressed at low levels compared to other cellular proteins. As a result, mass spectrometry-based proteomics may detect numerous irrelevant proteins, making it difficult to identify mechano- and CIP-sensitive TAFs.

Despite the numerous observations, it is evident that cell–cell contact alone does not instantaneously induce CIP. For instance, in Madin-Darby canine kidney (MDCK) cells, CIP occurs through three distinct phases: (i) a period of increasing cell density where cell movement gradually decreases, yet mitosis continues; (ii) a rapid shift to epithelial cell morphology; and (iii) ongoing cell division coupled with a reduction in cell size, accompanied by a progressively slower rate of mitosis. A halt in mitosis is reached once the cell area drops below a certain level [148]. Interestingly, the application of external stretching forces to cells under CIP can reinitiate the progression of the cell cycle [149]. In immortalized human mammary epithelial cells (MCF10A and MII cells) and HaCaT keratinocytes, confluent cells exhibit only about a 30% reduction in proliferation and only partial cytoplasmic localization of YAP1, whereas high-density cells fully stall proliferation [61]. These results suggest that contact between cells is not sufficient for inhibition of cell proliferation. Instead, CIP results from mechanical constraints that lead to a decrease in cell area with each successive cell division. Therefore, it appears to be true that “So far, there is no clear mechanistic connection between CIL and CIP, and they should not be thought of as interrelated processes as some people have suggested” [7]. However, recent evidence has revealed overlapping signaling pathways between CIL and CIP, as described earlier. To reconcile this apparent discrepancy, several possibilities can be considered. One possibility is that there might be a time lag between cell–cell contact and the full induction of CIP and/or the mechanical changes associated with high cell density. For instance, it has been observed that the size of the nucleus depends on the density of cells, and the size remains relatively constant even when cells come into contact with each other, indicating that NPC is still being stretched at confluency [61,87]. Another explanation could be that the duration of signaling defines the cell’s fate. It is plausible that prolonged signaling is necessary to fully switch from CIL to CIP. Perhaps more extended exposure to specific signaling cues is required to trigger the complete transition from cell migration to growth inhibition. Consistent with this concept, prolonged mechanical muscle loading results in an increase in the expression of mechanosensor genes and a switch in muscle fiber type [150].

In future research, it is essential to investigate the potential common and distinctive signaling pathways for CIP and mechanotransduction, as depicted in Figure 4. However, before exploring these pathways, it is necessary to identify all the key players, including sensors, transmitters, converters, and TAFs.

### 3.2. Nuclear Lamina, Mechanics, and Positioning

**Figure 5:**
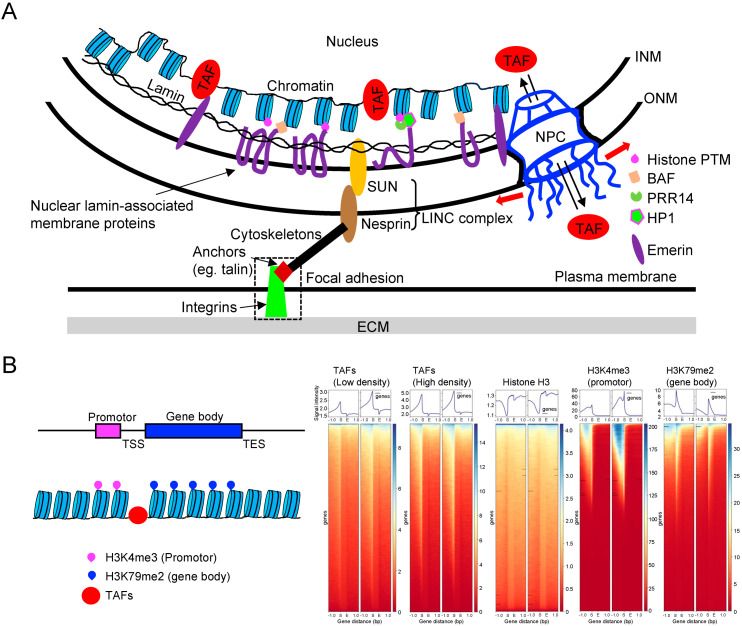
Regulation of chromatin structure and stretching of NPC in CI and mechanotransduction. (**A**) The physical connection between chromatin and the ECM may lead to the opening of chromatin regions where transcription-associated factors (TAFs) can bind for gene expression. This process could be regulated by the transmission of mechanical force through transmembrane proteins, the cytoskeleton, the LINC complex, and nuclear lamins. Additionally, the movement of TAFs could be influenced by the mechanical stretching of NPC. The SUN domains of SUN proteins directly interact with the KASH domains of nesprin proteins. Nuclear lamin-associated membrane proteins include emerin, LAP1/2, LBR, and MAN1 (inner nuclear membrane protein Man1). PTM: post-translational modification, HP1: heterochromatin protein 1. Numerous additional proteins have been detected in the nuclear envelope using proteomic methods, yet these findings require further confirmation [170]. (**B**) TAFs (red) bind to open chromatin to regulate gene expression. These binding loci of TAFs are distinct from the gene body, where modifications like histone H3 lysine-79 dimethylation (H3K79me2, blue) are present. Furthermore, histone H3 lysine-4 trimethylation (H3K4me3) modifications (promoter, magenta) are found in broader regions upstream of the transcription start site (TSS). TES, transcription end site. In various biological processes, such as CI and other mechanotransduction-mediated biological processes, gene regulation involves changes in chromatin structure due to epigenetic modifications. The DSP-MNase-proteogenomics method identifies TAFs and their binding loci [171]. The heatmaps represent the read coverage or signal intensities across genomic regions and DNA samples extracted from open chromatin using MNase (see Figure 6 for details). Heatmaps identify patterns of enrichment or depletion in specific regions of open chromatin and can provide insights into the regulatory landscape of the genome. Heatmap analysis of DNA fragments extracted from low- and high-density cells revealed that TAFs bind to accessible regions of chromatin, which are situated near the upstream of TSS, and displace histone H3 in the process. As expected, H3K4me3 and H3K79me2 modifications are enriched in the promoter region and gene body, respectively.

The nuclear lamina, depicted in Figure 5A, is composed of lamins and nuclear lamin-associated membrane proteins, forming a meshwork lining the inner surface of the nuclear envelope. Lamins are intermediate filaments that can be classified into A-type (lamin-A/C) or B-type (lamin-B1 and B2). The expression of lamin-A/C, but not lamin-B, correlates with tissue stiffness and is upregulated when cells are seeded on stiff substrates or subjected to stretching [78,151,152]. Conversely, culturing cells on a soft substrate or inhibiting myosin II reduces lamin-A/C levels, presumably by increasing their phosphorylation and solubilization [151]. Adherent cells express higher levels of lamin-A/C compared to non-adherent cells, underscoring the importance of lamin-A/C in mechanotransduction. Mutations in the LMNA gene lead to defective mechanotransduction and human diseases known as laminopathies. For instance, LMNA mutations causing muscular dystrophies increase YAP1 nuclear entry in muscle stem cells, even at high cell density [153]. Reduced expression of lamin A/C is also frequently associated with cancer phenotypes [154]. These mutations and decreased lamin A/C expression can induce genomic instability [155], potentially disrupting the normal association of the genome with the nuclear lamina. Dysfunction of lamin A/C would likely impact the stability of the genome, as lamina-associated domains (LADs) occupy about 30–40% of the total genome, and genes localized within LADs are typically transcriptionally silenced [156].

The LINC complexes play a crucial role in connecting chromatin to the cytoskeletons (Figure 5A) [157]. These LINC complexes consist of nesprin proteins (KASH: Klarsicht, ANC-1, Syne Homology proteins) located on the outer nuclear membrane (ONM) that bind to cytoskeletons. Nesprins, in turn, interact with SUN (Sad1p, UNC-84) proteins, which are linked to inner nuclear membrane (INM) proteins such as lamins, chromatin, and other nuclear envelope proteins. As a result, both external and internal forces are transmitted through the cytoskeleton to the nucleus, leading to changes in nuclear morphology and gene expression [158]. This force transmission to chromatin may influence DNA mechanics and structure, thereby regulating early transcription initiation events [159].

The nuclear lamin-associated membrane proteins are found embedded in or associated with the INM or lamins. Among these proteins, lamina-associated polypeptides 1 and 2 (LAP1, LAP2) and lamin B receptor (LBR) have been identified as INM transmembrane proteins that bind heterochromatin through the adapter protein [160]. Another INM transmembrane protein is emerin, which interacts with other INM proteins, including lamins, NPC proteins, and chromatin [161]. Moreover, emerin binds to several transcription regulators, such as ß-catenin and BAF (Barrier to Autointegration Factor). Notably, the loss of emerin has been shown to reduce mechanosensitive gene expression induced by strain stimuli [162], suggesting that emerin plays a role in mechanotransduction. In addition, the non-transmembrane protein PRR14 (proline-rich protein 14) also connects the nuclear lamina to histone H3 lysine-9 trimethylation (H3K9me3)-modified heterochromatin [163,164]. These findings suggest that lamin-associated proteins may play a role in mechanotransduction through cytoskeletons subjected to mechanical force, although a comprehensive characterization is still pending. Additionally, both lamin A/C and B have the ability to directly bind core histones through a specific sequence element in their tail domain [165].

The lamina and chromatin are the major determinants of nuclear mechanics. Although lamin A is a key element regulating nuclear shape and rigidity, heterochromatin is more densely packed and makes nuclei stiffer, whereas loose euchromatin is less stiff than compact heterochromatin [166]. Nuclear membrane curvature and tension are also important parameters in nuclear mechanics and can alter NPC to regulate the entry of YAP1, as discussed earlier. During cell movement, mitosis, and various cellular morphogenetic processes, such as the formation of muscle cells during development, nuclear movements occur. Dysregulation of nuclear positioning can result in functional impairments and contribute to disease [167]. Given that nuclear movements can impact the shape and mechanical properties of the nucleus, as well as alter lamin and NPC (Figure 5A), it is reasonable to consider that nuclear positioning plays a role in mechanotransduction and in the pathway of CI. In fact, the expression of dominant negative forms of the LINC complex components impairs force transmission from the cytoskeleton to the nucleus and impairs nuclear deformation [168]. However, recent discoveries indicate that the LINC complex may not always be essential for transmitting mechanical signals to the nucleus [169]. Nevertheless, the precise mechanism by which nuclear movement influences gene expression remains unclear.

## 4. TAFs in CIP and Downstream of Mechanotransduction

TAFs engage with cis-regulatory elements to regulate gene expression and comprise a wide range of molecules, such as RNA, heterogeneous nuclear ribonucleoproteins, and various protein factors. This review will primarily focus on TAF proteins that directly or indirectly bind to DNA, such as transcription factors (TFs), co-factors, repressors, and RNA polymerase [172], all of which are influenced during CIP and downstream of mechanotransduction. While researchers have identified more than 40 TAFs that shuttle between the nucleus and cytosol in response to cell–cell contact and mechanical stimulation (Table S1), our understanding of the changes occurring at the chromatin regions where these TAFs bind remains limited.

Chromatin is composed of DNA spooled around histones in a manner that dynamically exposes specific loci of DNA to TAFs that regulate transcription and replication by binding to the DNA (Figure 5B). To systematically profile chromatin, it is necessary to detect the genomic locations of these exposed loci and identify the TAFs bound to each locus. The former task is effectively accomplished using current techniques. For example, H3K4me3 modification is associated with gene expression and transcriptional activity, and DNA fragments associated with H3K4me3 can be sequenced and mapped on the genome through chromatin immunoprecipitation with sequencing (ChIP-seq) using anti-H3K4me3 antibodies and next-generation sequencing (NGS) technologies [173,174]. Similarly, DNA fragments attached to H3K79me2 are associated with gene bodies [175] (Figure 5B), and ChIP-seq analysis using anti-histone H3 antibodies reveals that upstream regions near the transcription start site (TSS) of many genes lack histone H3, suggesting that these regions may interact with TAFs.

**Figure 6:**
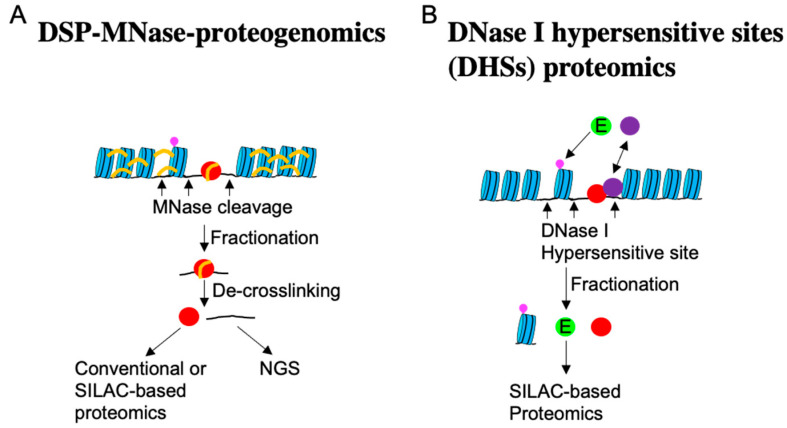
Methods to identify mechanosenstive TAFs. (**A**) DSP-MNase-proteogenomics [171]. DNA-TAF complex is stabilized with a reversible cross-linker, dithiobis(succinimidyl propionate) (DSP). After washing out the majority of proteins, the DNA-TAF complex is cleaved off using micrococcal nuclease (MNase) and separated from cross-linked chromatin by centrifugation. After de-cross-linking the cleaved DNA-TAF complex, TFA proteins can be identified by conventional or stable isotope labeling by amino acids in cell culture (SILAC)-based proteomics. DNA fragments can be sequenced by NGS. (**B**) DNase I hypersensitive sites (DHSs) proteomics [87]. After washing out soluble cytoplasmic proteins using hypotonic buffer, loose and open chromatin is cleaved off using DNase I and fractionated. Proteomics using SILAC quantitatively identify proteins that increased or decreased after stimulation. Since no crosslinking and extensive washing are involved, proteins that transiently associate with chromatin can be detected. Light blue indicates nucleosome. Red (strong interaction) and purple (transient interaction) indicate TAFs. Green indicates enzyme. Magenta indicates PTM of histone. Orange indicates DSP crosslinker.

Recently, a method to comprehensively determine TAFs and their binding loci on chromatin (DSP-MNase-proteogenomics) has been developed [171] (Figure 6).

Through this method, new nucleocytoplasmic shuttling molecules sensitive to cell density, such as PTGES3, TIPARP, SMAD4, and CBFB (Table S1), have been discovered. Moreover, DSP-MNase-proteogenomics revealed that DNA fragments binding to TAFs are notably enriched upstream of the TSS (Figure 5B), as anticipated. This analysis enables the comparison of active genes across multiple samples and has shown that genes associated with the Hippo pathway are downregulated in high-density cells [171]. Another method known as DNase I hypersensitive sites (DHSs) proteomics, described in Figure 6 [87], also facilitates the quantitative comparison of multiple samples and the identification of TAFs that transiently interact with chromatin. Using this approach, UBE2A/B, which demonstrates nucleocytoplasmic shuttling depending on cell density, substrate stiffness, and drug-induced inhibition of myosin contraction, has been identified. Similar to YAP1, UBE2A/B relocates to the nucleus when cells are cultured at low density and on a stiff substrate, where it ubiquitinates histone 2B Lys120. Knocking down UBE2A/B leads to the suppression of many genes, including YAP1 [87].

Various methods are employed to investigate the nucleocytoplasmic shuttling of TAFs, including photo-perturbation-based techniques such as fluorescent recovery after photo-bleaching, fluorescence correlation spectroscopy, and single-particle tracking. However, it is important to note that each of these methods has its own set of advantages and limitations [176]. When green fluorescent protein (GFP) is genetically attached to a protein of interest, the behavior of the exogenous epitope-tagged protein can be misleading [177]. However, in some cases, when the GEP gene is knocked-in to tag the gene of interest into genomic DNA, it appears to replicate the behavior of endogenous proteins [56]. Nevertheless, some groups could demonstrate that overexpressed GFP-YAP1 behaves like endogenous YAP1 [62,178].

## 5. Ubiquitination: A Key Mechanism in the Pathways of CIP and Downstream of Mechanotransduction

Ubiquitination serves a crucial role not only in protein degradation but also in various other biological processes, including DNA repair, activation of protein kinases, and transcription [179,180,181,182,183,184]. Additionally, ubiquitination has been implicated in CI and mechanotransduction [87]. For example, pressure overload and hypertrophy of cardiomyocytes enhance ubiquitination, where beta 3 integrin mediates the mechanotransduction [185]. When increased mechanical tension develops in intact smooth muscle, Skp2, a component of a ubiquitin ligase complex, mediates the degradation of several proteins that inhibit proliferation [186]. The chaperone-assisted selective autophagy complex, comprised of Hsc70, HspB8, and BAG3, senses the mechanical unfolding of filamin. Together with the chaperone-associated ubiquitin ligase CHIP, the complex initiates the ubiquitin-dependent autophagic sorting of damaged filamin to lysosomes for degradation to maintain homeostasis [187]. These findings strongly suggest that ubiquitination plays a significant role in mechanotransduction. However, the precise mechanisms by which ubiquitination regulates these diverse biological processes remain unclear. Nevertheless, some evidence indicates that mechanical forces induce the ubiquitination of YAP1, which is implicated in tumorigenesis and angiogenesis [188,189,190,191].

The expression of beta-catenin, cadherin, and ubiquitin domain-containing protein 1 (UBTD1) increases in response to cell density [192]. UBTD1 localizes at cell junctions on a stiff substrate, while it mostly remains diffused in the cytoplasm on a soft substrate. Inhibition of actomyosin contraction, or actin depolymerization, also promotes the delocalization of UBTD1 from the membrane to the cytosol. In the cytoplasm, UBTD1 associates with the YAP1 degradation complex, which comprises UbcH5, b-TrCP, and beta-catenin. This association facilitates YAP1 ubiquitylation for proteasomal degradation. Interestingly, at low cell density, UBTD1 is weakly expressed, leading to RhoA activation and myosin contraction, promoting nuclear localization of YAP1. These findings are consistent with observations in hepatocellular carcinoma cells, where UBTD1 expression significantly decreases with increasing matrix stiffness [188].

Ubiquitination of YAP1 is also triggered when mouse fibroblasts are cultured at high density, resulting in polyubiquitination and subsequent degradation of YAP1 through a proteasome-dependent pathway [193]. However, a different study has demonstrated that when human umbilical vein endothelial cells are exposed to pulsatile flow, ubiquitination and degradation of YAP1 occur independently of the proteasome [194].

The protocadherin FAT1 exerts a negative regulatory effect on endothelial cell proliferation during angiogenesis by promoting the E3 ubiquitin ligase Mind Bomb-2 (MIB2)-mediated ubiquitination and degradation of YAP1 [191]. Such ubiquitinated YAP1 could be attenuated by herpes virus-associated ubiquitin-specific protease (HAUSP)-mediated deubiquitination [195]. Moreover, YAP1 undergoes non-proteolytic, K63-linked polyubiquitination by the SCFSKP2 E3 ligase complex (SKP2), promoting its nuclear localization and transcriptional activity independently of Hippo pathway-mediated phosphorylation of YAP1 [196]. This ubiquitination process is reversed by the deubiquitinase OTUD1. In each of these cases, the level of ubiquitination appears to be quite low, as detected by Western blotting against YAP1, where the ubiquitinated form is barely visible at a high molecular weight. It is possible that ubiquitination is transiently required in non-proteolytic pathways and leads to rapid degradation in proteolytic pathways.

UBE2A/B functions as an E2 ubiquitin-conjugating enzyme, capable of accepting ubiquitin from the E1 complex and transferring it to other proteins [197]. Ubiquitinated proteins can be detected using anti-ubiquitin antibodies with varying specificities, some recognizing mono- or polyubiquitin, linear or branched ubiquitin, or site-specific ubiquitin. Interestingly, when cells are stained with the F-11 monoclonal antibody (Santa Cruz Biotechnology, sc-271289), which targets ubiquitin and polyubiquitin, the nucleus exhibits intense staining in low-density cells, whereas the cytosol is strongly stained in high-density cells [87]. As this antibody reacts with a wide range of proteins in Western blotting, this result suggests the presence of numerous unidentified ubiquitinated proteins that shuttle between the nucleus and cytosol during CI. However, the anti-ubiquitin P4D1 monoclonal antibody (Santa Cruz Biotechnology, sc-8017) did not exhibit such a difference in staining, indicating that CI-dependent translocation may be contingent on the conformation of the ubiquitin chain(s).

## 6. Autophagy in CIP and Mechanotransduction

In cells situated at high density or on a soft substrate, phosphorylated YAP1 relocates to the cytosol, leading to decreased transcription of actomyosin genes (MLC2, MYH10, and MYH14), resulting in reduced formation of F-actin stress fibers. Consequently, this impairs autophagosome formation, leading to a reduction in both cell proliferation and basal autophagy [198]. Conversely, at low cell density, YAP1 translocates into the nucleus, activating the expression of actomyosin genes, which induces the formation of actin stress fibers and maintains YAP1 in the nucleus. The mechanical strain exerted on mesenchymal stem cells activates mTORC2, a regulator of autophagy that influences various downstream effectors [199]. This mechanical activation of mTORC2 can be prevented by pharmacological inhibition of focal adhesion kinase (FAK), which is mechanosensitive. FAK is recruited to focal adhesion sites upon cell attachment and mechanical strain [200]. Other force-induced signaling molecules, such as ions and kinases, and the ECM also play roles in regulating autophagy [201]. Conversely, autophagy itself regulates mechanotransduction by recycling cellular components, providing energy, and contributing to ECM secretion [202].

## 7. FLNA-Mediated Mechanotransduction and Its Potential Role in CIL and CIP

While lysed cells and reconstituted systems have provided valuable insights into certain cellular mechanisms, the incorporation of mechanical force as a parameter to simulate physiological conditions has been largely overlooked, despite its fundamental importance. Reconstituting internal or external mechanical stress in vitro while simultaneously quantifying protein–protein interactions remains a significant challenge, hindering progress in this area of research. To surmount this obstacle, gaining a comprehensive understanding of structural details becomes crucial to strategically designing a probe that can mimic the mechanically activated conformation of a molecule. Among the molecules known to mediate mechanotransduction, FLNA stands out as one of the best-characterized examples [99,203].

**Figure 7:**
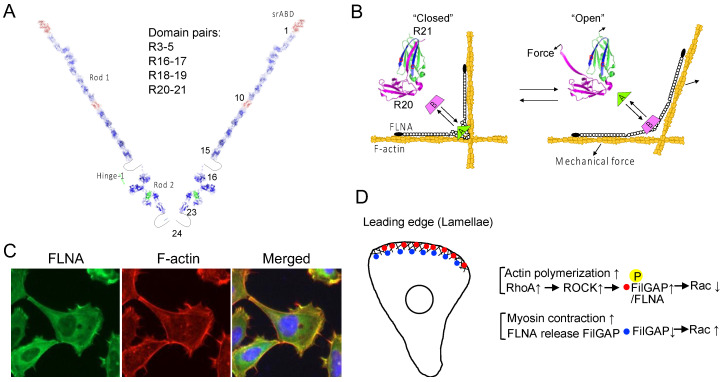
FLNA-mediated mechanotransduction. (**A**) The structure of FLNA includes paired domains that may undergo unfolding in response to mechanical forces [99,203]. (**B**) Mechanical force induces conformational changes in the FLNA molecule, exposing cryptic binding sites and altering the geometry of FLNA subunits (e.g., A: FilGAP, B: integrins). (**C**) FLNA and F-actin are highly concentrated at the leading edge of the cell. 100 × 100 μm. (**D**) The model proposes that cell protraction at the leading edge is regulated by the force-dependent interaction between FLNA and FilGAP. The yellow-highlighted “P” indicates phosphorylation (activated FilGAP). For further details, refer to the text.

Mutations and loss of FLNA genes are associated with a wide range of human diseases and alter cell mechanics and behaviors [99,204]. FLNA is a homodimerized actin cross-linking protein. Each subunit has an N-terminal spectrin-like actin-binding domain (srABD) that consists of two calponin homology domains, followed by 24 immunoglobulin-like repeats (R). Two unstructured hinges (32 amino acid residues) separate the subunit into three segments, namely rod-1 (R1–15), rod-2 (R16–23), and the dimerization domain (R24) (Figure 7A). Each repeat consists of seven β-strands (A–G) in parallel orientation. Interestingly, some of the repeats form domain pairs with unique characteristics. For instance, R24 utilizes strands C and D to create an antiparallel β sheet through hydrogen bonding and hydrophobic interactions. In contrast, other domain pairs exhibit different interactions. For instance, FLNA R3 and R4 interact through the edges of the β sheets, while R4 and R5 interact across three β-strands on each side, resulting in an antiparallel domain pair of R3–5 that stabilizes R4 (protein data bank (PDB): 4M9P) [205]. Furthermore, because β-strand A of FLNA R16 does not fold as part of R16, exposed hydrophobic β-strands on the BG face bind tightly to the AG face of R17, and the free strand A of R16 could provide additional flexibility in hinge-1 (PDB: 2K7P). Importantly, this domain pair does not interfere with the interaction between the cytoplasmic tail of GPIbα and the CD face of R17 [206]. The β-strands A of FLNA R18 and R20 also remain unfolded, instead covering the CD faces of R19 and R21, respectively, where the cytoplasmic domain of β integrins binds (Figure 7B). As a result, while the integrin β7 peptide binds to R19 and R21 independently, it does not interact with R18-19 (PDB: 2K7Q) or R20-21 (PDB: 2J3S). These domain pairs contribute to compacting the rod-2 segment [207,208], which can be unfolded by mechanical forces [99,100,141,209].

The force needed to unfold the domain pairs has been measured at approximately 10 pN or even less, while the repeats in the rod 1 segment and dimerization domain can withstand significantly higher forces [210,211].

Given that a single myosin molecule can generate 3–4 pN force, just one or a few myosins can produce enough force to unfold the domain pairs [100,141], allowing for partner interactions. So far, several molecules, including β integrins, migfilin, CFTR, fimbacin, smoothelin, G3BP1, SAV1, and LARP4, have been shown to interact with the CD face of R21 [99,212,213,214,215,216]. This suggests that these molecules interact with FLNA in a force-dependent manner. While the intracellular concentration of FLNA seems sufficient for interactions with all these binding partners (e.g., FLNA: 6 μM and G3BP1: 0.6 μM) [214], it’s worth noting that the concentrations of many signaling molecules involved have not been measured and may vary in different cellular regions. Furthermore, there is a possibility that other domain pairs could be mechanosensitive and engage with some of these signaling molecules due to the similarity between the CD face of R21 and that of R4, R9, R12, R17, R19, and R23. Nevertheless, as of now, no partner protein that interacts with these domains in a force-dependent manner has been identified, except for R23.

In FLNA dimers, the two R23 domains are spatially separated due to the dimerization of R24 and hinge-2 [207]. FilGAP, a filamin-binding Rac-specific GTPase-activating protein, also homodimerizes through its C-terminal coiled-coil domain, providing two FLNA-binding sites [217]. In a relaxed state, FilGAP interacts with R23 through each FLNA subunit [218]. However, the application of mechanical force can cause the separation of the two R23 domains, leading to a decrease in their avidity, ultimately releasing FilGAP from its interaction with FLNA [100]. Unlike R19 and R21, the CD face of R4, R9, R12, and R17 is not covered with the strand A of their preceding repeats; however, it is possible that these repeats might exhibit mechanosensitivity in a manner similar to that of R23.

## 8. FLNA Potentially Regulates CIL through FilGAP

FLNA is enriched in the lamellae of the leading edge of migrating cells (Figure 7C). Additionally, FilGAP localizes to the cell front via its pleckstrin-homology domain in a phosphatidylinositol 3,4,5-trisphosphate (PtdIns(3,4,5)P3)-dependent manner [219]. FilGAP can be activated through ROCK-mediated phosphorylation, in a process dependent on FLNA. As a result, the force-dependent interaction between FLNA and FilGAP may play a role in regulating FilGAP activity and controlling Rac1 activity.

In the traditional view, Rac1 dominates RhoA at the leading edge and oppositely at the rear to maintain polarity and directed migration [220]. Further detailed analysis revealed that RhoA is activated at the cell edge, while Rac1 activation occurs 2 μm behind the edge with a 40 s delay in randomly migrating cells [221]. These observations suggest that RhoA initiates protrusion, whereas Rac1 and Cdc42 reinforce and stabilize newly expanded protrusions. This spatial restriction and delay could be regulated by FilGAP, which is associated with the plasma membrane through PtdIns(3,4,5)P3, thus maintaining Rac1 in an inactive state [222] (Figure 7D). FilGAP phosphorylation by ROCK and its binding to FLNA could further contribute to Rac1 inactivation at the cell edge [217]. Therefore, it is possible that the decrease in traction forces at cell–cell contacts due to CIL [223] leads to the stalling of Rac recycling. Moreover, actomyosin contraction, triggered by RhoA behind the cell edge, would lead to the dissociation of FilGAP from FLNA, thereby inactivating FilGAP and activating Rac1. 

## 9. FLNA Regulates CIP through the Hippo Pathway

Involvement of *Drosophila* filamin (cheerio) in the Hippo pathway to induce hypertrophic growth has been demonstrated [224]. However, the molecular mechanism by which cheerio controls the Hippo pathway remains unknown. Recently, SAV1, a component of the Hippo pathway, has been identified as a binding partner of FLNA in mammalian cells [215]. Like other known mechanobinding partners of FLNA, SAV1 interacts with the CD face of R21. When myosin contraction is lost, SAV1 is released from cytoplasmic FLNA and partially diffuses to the nucleus. This suggests that mechanical force may regulate the localization of YAP1 in a Hippo-dependent manner, mediated by force-induced conformational changes of FLNA. To investigate this hypothesis, conditional transgenic mice expressing non-FLNA-binding Sav1 were generated in the liver [225]. However, this experiment encountered an unexpected issue, as the insertion of the flox cassette led to exon 2 skipping, resulting in the deletion of exon 2. Since exon 2 encodes the FLNA-binding site, the mutant Sav1 is unable to bind to FLNA. Interestingly, disruption of the FLNA-SAV1 interaction in these mice led to growth retardation, accompanied by apoptosis induced by an as-yet-unknown mechanism. As the deletion of exon 2 could potentially affect other functions of SAV1, a more targeted inhibition of the FLNA-SAV1 interaction is essential to definitively validate or refute the hypothesis.

**Figure 8:**
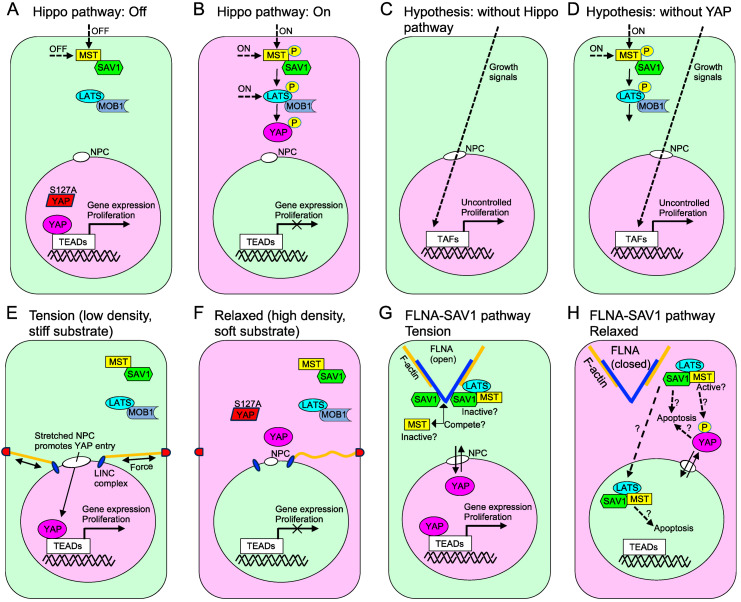
Hippo-dependent and -independent pathways for cell proliferation. The pink-colored nucleus indicates the localization of YAP1/TAZ in the nucleus, suggesting a cell growth state. Nevertheless, it has been reported that YAP1 nuclear localization alone is insufficient as an indicator of YAP1 activity (see Section 3). (**A**,**B**) The Hippo pathway can be turned off or inhibited by several mechanisms and factors, such as alterations in cell polarity, growth factors, oncogenic signaling, nutrient availability and metabolic signals. When the Hippo pathway is turned off, the core kinases of the pathway, particularly LATS1/2, become inactive. This leads to reduced or no phosphorylation of YAP1. Non-phosphorylated YAP1 then translocates to the nucleus and acts as a transcriptional co-activator. It binds to various transcription factors, most notably those in the TEAD family. This interaction triggers the activation of genes that promote cell proliferation and survival (resistance to apoptosis), as well as other genes involved in processes such as stem cell activation. The S127A mutant of YAP1 is resistant to phosphorylation and is retained within the nucleus [230]. The Hippo pathway is activated by various extracellular and intracellular signals, including G-protein-coupled receptor signaling, as well as signals downstream of cellular metabolism and CIP. These signals can influence the activity of the pathway’s core components, which include mammalian STE20-like protein kinases (MST1/2) and LATS1/2. SAV1, MOB1, and other factors also promote the kinase activity of their binding partners. Once activated, LATS1/2 phosphorylate YAP1/TAZ. When phosphorylated, YAP1/TAZ are retained in the cytoplasm and become subject to degradation. Normally, the Hippo pathway restricts growth by suppressing YAP1. However, oncogenes such as Ras and Src can either inhibit the Hippo pathway or directly activate YAP1, thus promoting uncontrolled cell growth and contributing to cancer development. (**C**,**D**) Hypothesis: Cells can proliferate through various growth signals even in the absence of the Hippo pathway or YAP1 (this can occur even when the Hippo pathway is active), potentially leading to uncontrolled growth. (**E**,**F**) Mechanical forces can influence YAP1 translocation into the nucleus independently of the Hippo pathway. YAP1 remains in the cytosol on soft substrates, even when its phosphorylation is inhibited, indicating that dephosphorylation alone does not ensure its nuclear entry. The stretching of NPC through mechanical forces and substrate stiffness, mediated by the LINC complex and actin cytoskeleton, plays a key role in YAP1’s nuclear import, which varies with substrate rigidity [62]. Nevertheless, it has been observed that the opening of the NPC alone is insufficient for the nuclear entry of YAP1 (see Section 3). (**G**,**H**) The binding of SAV1 to FLNA under tension retains MST1/2 and LATS1/2 in the cytoplasm, thereby endorsing the activity of YAP1 in the nucleus. In relaxed cells, SAV1 is released from FLNA to induce apoptosis by an unknown mechanism. Some of the released SAV1 might translocate to the nucleus, presumably with MST1/2 and LATS1/2. Phosphorylation of YAP1 might take place in the cytosol if non-phospho-YAP1 can exit the nucleus [225].

The expression of YAP1 S127A, which is not affected by Hippo pathway phosphorylation and localizes in the nucleus, led to enlarged livers, as anticipated (Figure 8A) [226,227]. However, contrary to conventional expectations, the depletion of YAP1/TAZ in mouse livers surprisingly resulted in larger liver size compared to their wild-type counterparts, although liver regeneration is less efficient when YAP1/TAZ is deleted [228,229]. This finding suggests that YAP1/TAZ are not essential for liver growth per se but rather act as potent inducers of cell proliferation in the nucleus and as an inhibitor for overgrowth, regulated by the Hippo pathway (Figure 8B–D) [230]. During the early phases of evolution, there is a belief that single-celled organisms could continuously multiply under favorable conditions. In fact, numerous single-celled organisms currently exhibit these traits. However, in the transition from single-celled to multicellular organisms with distinct morphology, it is presumed that CIP and apoptosis were necessary. In other words, it is conceivable that the Hippo pathway is not crucial for cell proliferation but rather primarily functions to hinder cell proliferation in specific-sized organisms. The suppression of this pathway, whether triggered by mechanical or biochemical stimuli or induced by diseases, fosters cell proliferation. Taken together, one could hypothesize that the enlargement of the liver upon loss of YAP1/TAZ is attributed to the absence of a SAV1 effector, as SAV1 is known to inhibit liver growth [231,232]. It is possible that SAV1 released from FLNA by reduced mechanical force may not effectively suppress proliferation without the presence of SAV1’s effector (cytosolic YAP1/TAZ), which may have an apoptotic or inhibitory activity to control overgrowth. Nevertheless, there has been relatively little exploration of the function of cytosolic YAP1/TAZ (Figure 8E–H).

## 10. Cell and Tissue Mechanics in Normal and Aberrant Physiology

Human tissues are subjected to a diverse range of mechanical forces, including flow shear, compression, tension, osmotic pressure, myosin-mediated contraction, and gravitational forces [13,14]. Moreover, the mechanical properties of tissues themselves play a crucial role in cell growth, stem cell differentiation, cancer development, and even Alzheimer’s disease [16,233,234,235]. Additionally, diseases can also alter the mechanical properties of cells themselves. For instance, fibroblasts from Dupuytren’s disease exhibit increased stiffness compared to normal fibroblasts [236]. Conversely, highly invasive cancer cells often display softer mechanical properties with distinct lamellipodial and protrusive structures, facilitating cell deformation and shape changes necessary for invasion [237].

Less invasive cell lines tend to exhibit higher stiffness, likely due to increased cortical actin and phosphorylated active myosin light chains. However, cancerous tissues, in general, are stiffer than normal tissues, mainly attributed to the increased stiffness of the stroma [238] (Table S2). This increased stromal stiffness is caused by the accumulation of ECM components, ECM crosslinking, and fibroblast proliferation within cancer tissues [238]. It is noteworthy that oncogene activity alone is insufficient for the oncogenic reprogramming of normal cells, but supraphysiological ECM rigidity can promote tumorigenesis [46,239]. However, different studies suggest that cellular responses to ECM stiffness are influenced by the dimensionality (two-dimensional or three-dimensional, 2D or 3D, respectively) and degradability of the ECM [240,241,242]. For instance, cell volume increases with high ECM stiffness in 2D but decreases in 3D, whereas cells in 3D hydrogels with protease-degradable crosslinks exhibit higher cell volume and nuclear YAP1 localization compared to non-degradable ECM, likely due to improved cell spreading [241]. These results indicate that cell mechanics alone is not the sole determinant of cell fate, and the localization of specific molecules, such as YAP1, does not solely dictate cell fate. Additionally, isolated neonatal mouse cardiomyocytes show higher proliferation on soft substrates compared to stiff substrates, further supporting the idea that the microenvironment plays a critical role in cell behavior [243]. Another important characteristic of the ECM is its ability to dissipate sustained stresses, such as continuous pressure and touch, providing protection to the cells from these mechanical forces. On the other hand, rapid deformations of the ECM can be sensed by cells that are connected to the ECM [244]. Furthermore, when cells attach to the ECM through specific sites such as focal adhesions, this attachment can define the overall shape of the cells, which, in turn, influences their behavior and function [245].

Cell mechanics are influenced not only by the interaction with the ECM but also by the mechanics of the cytoskeleton within the cells. Motor proteins play a crucial role in mechanically stressing actin filaments and microtubules, causing their binding proteins and the filaments themselves to undergo mechanical deformation, which can lead to changes in their functions [246,247]. Other cytoskeleton-associated proteins that mediate polymerization, depolymerization, cross-linking, bundling, and severing also alter the mechanics of the cytoskeleton [32,33]. Intermediate filaments, including nuclear lamins and vimentins, are connected to other cytoskeletal elements through the LINC complex and binding proteins like plectin. Therefore, intermediate filaments may also play a role in mechanotransduction, but their molecular mechanism is less explored compared to other cytoskeletal components [248].

Stiffness, also referred to as rigidity or elastic modulus, characterizes the ability of a material to resist deformation when subjected to a force applied at a slow rate. In elastic deformation, the strain is reversible, meaning the material returns to its original shape after the force is removed. On the other hand, cells and tissues can undergo irreversible changes in their shapes through ductile deformation, during which molecular bonds may break but can be reformed into new bonds through plastic or viscous flow (viscoelastic). The stiffness of human tissues varies significantly, ranging from 0.4 to 1.4 kPa in the brain to 8 to 40 GPa in bone. These variations are larger than the differences observed between the stiffness of normal and abnormal tissues (Table S2). This suggests that different types of cells possess unique thresholds that distinguish between normal and abnormal states, growth and growth arrest, or differentiation and proliferation.

Biological materials exhibit nonlinear elasticity, meaning they are soft under low strain and stiffen under higher strain (strain-stiffening) to protect cells and tissues from large deformations [249]. However, the precise relationship between strain and the mechanical activation of molecules involved in cellular mechanotransduction and cell-ECM interaction remains poorly understood. For instance, the softness of actin networks cross-linked by FLNA under low strain can be attributed to the semi-flexible nature of the FLNA molecule, while stiffness under higher strain (strain-stiffening) is driven by the strong avidity of the FLNA-F-actin interaction [207]. The plasticity of cells and tissues is influenced by time-dependent biomolecular interactions. For example, actin networks crosslinked by FLNA exhibit elastic behavior when the deformation rate is faster than the rate of crosslink exchange, but they behave as a viscous material when deformation is slow enough to allow crosslinker molecules to rearrange, resulting in a viscoelastic response [250]. Although it is likely that mechanical activation of FLNA occurs at high strain, the precise relationship between FLNA activation and the deformation of actin networks has not been thoroughly investigated. Various methods exist to measure the nanomechanics of individual proteins, as well as the viscoelastic properties of biopolymers, cells, and tissues, and to manipulate these properties for the study of CI and mechanotransduction [251,252,253,254,255]. Despite these advancements, there is still a need for further research to develop a comprehensive theory that can establish a connection between the measured mechanical properties and their impact on biological phenotypes.

If mechanical forces regulate cell growth, could the manipulation of such forces control an animal’s body size? For instance, might microgravity influence body dimensions in space? Internal mechanical forces are produced through the polymerization of cytoskeletal proteins and motor proteins. However, controlling these forces responsible for mechanotransduction without impacting other cellular processes, including cytokinesis and cell shape change, is impossible. Therefore, investigating the effect of internal mechanical forces on mechanotransduction-mediated cell growth is unfeasible. Given that external forces would impact internal forces, as discussed earlier, it is likely that the external forces would influence other cellular processes responsible for cell growth as well. Although it is well known that atrophy and bone absorption occur in microgravity, it appears that body size does not significantly change [256,257], presumably because animal cells and tissues regulate internal forces to adapt to an environment. However, it is intriguing to note that mice exposed to 37 days of spaceflight displayed elevated liver mass (33%) compared to ground-control mice [256].

## 11. Conclusions and Perspectives

In 2003, a seminal review article highlighted the significance of mechanical forces in both normal physiology and pathology [258]. Although this article presented several instances of mechanical force involvement in biological processes, the precise molecular mechanism underlying how these forces are sensed and translated into biochemical signals remained unclear at the time. Over the past two decades, the field has made considerable progress, and more recent review articles have been published focusing on mechanotransduction in human diseases [17,18,259,260,261,262]. As presented in Table S1, numerous cell density- and mechano-sensitive proteins have been identified, and ongoing research is exploring the molecular regulation of these molecules. Furthermore, the involvement of other biological pathways, such as metabolism (ATP synthesis and glycolysis) and Rho signaling, in CI and mechanotransduction has been discovered [263,264,265,266,267,268]. These findings have already led to the development of novel treatment strategies for certain diseases [15,269,270]. For instance, in some cases, reducing ECM stiffness using lysyl oxidase inhibitors has improved T cell migration and enhanced the effectiveness of anti-PD-1 treatment [271]. However, it did not yield similar results in idiopathic pulmonary fibrosis [272]. Additionally, the regenerative potential of tissues, such as the heart, can be enhanced by employing mechanically compliant ECM [273]. Despite these advancements, targeting specific mechanical aspects may have unintended negative consequences due to the intricate and multimodal nature of mechanotransduction pathways, as well as the complexity of multicellular and multidimensional forces present in tissues. Mechanical perturbations can trigger various gene expressions and post-translational modifications, making it challenging to pinpoint definitive causative factors as these changes might represent consequences rather than causes. Moreover, replicating mechanical factors in reconstitution systems poses a significant obstacle to advancing mechanotransduction research. A case in point is FLNA, where a thorough understanding of molecular mechanisms has enabled the rational design of molecules mimicking mechanically active forms. However, such examples are limited, and much remains to be explored. For instance, the generation of model animals lacking specific mechanotransduction pathways is relatively rare, partly because transgenic animals may compensate for the defect by bypassing the signaling pathway. Therefore, continued research and a deeper understanding of mechanotransduction mechanisms are essential to developing effective therapeutic interventions and fully unraveling the complex interplay between mechanical forces and biological processes. Lastly, it is worth mentioning that various cell types exhibit distinct responses to hyper-gravity or micro-gravity conditions [274,275]. For example, microgravity exposure during spaceflight causes the disordered regulation of liver function, presumably via regulation of YAP/TAZ activation [276]. Understanding how living organisms on Earth and in space utilize mechanotransduction to adapt to gravity is a crucial question, especially in the era of space travel.

In conclusion, the following questions in these fields warrant further investigation:

### 11.1. Molecular and Cellular Mechanisms

Identification of Key Molecules: Continue identifying and characterizing key molecules involved in mechanotransduction, CIL, and CIP, aiming to uncover both common and distinctive components.

Signaling Networks: Elucidate the signaling networks that orchestrate the cellular responses in each process in complex environments and investigate how they converge or diverge. How do cells adapt to mechanical stimuli over time, and is there a memory effect in mechanotransduction?

### 11.2. In Vivo and Physiological Relevance

Physiological Relevance: What is the physiological relevance of mechanotransduction, CIL, and CIP in complex in vivo environments, such as during tissue development, immune response, or wound healing? Investigate tissue-specific variations in mechanotransduction, CIL, and CIP to uncover context-dependent roles and responses.

In Vivo Models: Develop and utilize sophisticated in vivo models that recapitulate physiological conditions to understand the relevance of mechanotransduction, CIL, and CIP in complex biological systems.

### 11.3. Disease Connections and Clinical Applications

Disease Mechanisms: Explore the specific roles of mechanotransduction, CIL, and CIP in various diseases, aiming to identify disease-specific mechanisms and vulnerabilities.

Therapeutic Strategies: Investigate the development of therapeutic strategies targeting mechanotransduction, CIL, and CIP for diseases associated with aberrant cell responses.

Other Clinical Applications: Exploring the potential of targeting mechanotransduction, CIL, and CIP for other clinical applications such as tissue engineering, drug screening, diagnosis, and imaging.

### 11.4. Technological Advancements

Development of Innovative Tools: Developing novel tools and techniques for precise measurement and manipulation of mechanical forces at the cellular and molecular levels is an ongoing challenge. 

Single-Cell Analysis: Leverage single-cell analysis technologies to dissect heterogeneity in cell responses within populations, shedding light on individual cell behaviors in the context of mechanotransduction, CIL, and CIP.

Computational Modeling and Systems Biology: Develop comprehensive computational models that integrate data from mechanotransduction, CIL, and CIP to simulate and predict cellular behaviors under different conditions. In addition, apply systems biology approaches to understand the global impact of mechanotransduction, CIL, and CIP on cellular processes and functions.

By pursuing these future directions, researchers can advance our understanding of the intricate mechanisms governing cellular responses to mechanical cues and CI, paving the way for innovative therapies and applications in various fields.

## Data Availability

Not applicable.

## Supplementary Materials

The following supporting information can be downloaded at: https://www.mdpi.com/article/10.3390/ijms25042135/s1. References [277,278,279,280,281,282,283,284,285,286,287,288,289,290,291,292,293,294,295,296,297,298,299,300,301,302,303,304,305,306,307,308,309,310,311,312,313,314,315,316,317,318,319,320,321,322,323,324,325,326,327,328,329,330,331,332,333,334,335,336,337,338,339,340,341,342,343,344] are cited in the supplementary materials.

## References

[1] Abercrombie M., Heaysman J.E. (1953). Observations on the social behaviour of cells in tissue culture. I. Speed of movement of chick heart fibroblasts in relation to their mutual contacts. Exp. Cell Res..

[2] Roycroft A., Mayor R. (2018). Michael Abercrombie: Contact inhibition of locomotion and more. Int. J. Dev. Biol..

[3] Abercrombie M., Heaysman J.E. (1954). Observations on the social behaviour of cells in tissue culture. II. Monolayering of fibroblasts. Exp. Cell Res..

[4] Stoker M.G., Rubin H. (1967). Density dependent inhibition of cell growth in culture. Nature.

[5] Abercrombie M., Heaysman J.E., Karthauser H.M. (1957). Social behaviour of cells in tissue culture. III. Mutual influence of sarcoma cells and fibroblasts. Exp. Cell Res..

[6] Ribatti D. (2017). A revisited concept: Contact inhibition of growth. From cell biology to malignancy. Exp. Cell Res..

[7] Stramer B., Mayor R. (2017). Mechanisms and in vivo functions of contact inhibition of locomotion. Nat. Rev. Mol. Cell Biol..

[8] Jain H.P., Wenzel D., Voigt A. (2022). Impact of contact inhibition on collective cell migration and proliferation. Phys. Rev. E.

[9] Ahmad U.S., Uttagomol J., Wan H. (2022). The Regulation of the Hippo Pathway by Intercellular Junction Proteins. Life.

[10] Mammadova-Bach E., Gudermann T., Braun A. (2023). Platelet Mechanotransduction: Regulatory Cross Talk Between Mechanosensitive Receptors and Calcium Channels. Arterioscler. Thromb. Vasc. Biol..

[11] Young K.M., Reinhart-King C.A. (2023). Cellular mechanosignaling for sensing and transducing matrix rigidity. Curr. Opin. Cell Biol..

[12] Pillai E.K., Franze K. (2023). Mechanics in the nervous system: From development to disease. Neuron.

[13] Nakamura F. (2017). Mechanotransduction in blood cells. Asia-Pac. J. Blood Types Genes.

[14] Liu X., Nakamura F. (2020). Mechanotransduction, nanotechnology, and nanomedicine. J. Biomed. Res..

[15] Guo T., Wantono C., Tan Y., Deng F., Duan T., Liu D. (2023). Regulators, functions, and mechanotransduction pathways of matrix stiffness in hepatic disease. Front. Physiol..

[16] Saraswathibhatla A., Indana D., Chaudhuri O. (2023). Cell-extracellular matrix mechanotransduction in 3D. Nat. Rev. Mol. Cell Biol..

[17] Ezzo M., Hinz B. (2023). Novel approaches to target fibroblast mechanotransduction in fibroproliferative diseases. Pharmacol. Ther..

[18] Di X., Gao X., Peng L., Ai J., Jin X., Qi S., Li H., Wang K., Luo D. (2023). Cellular mechanotransduction in health and diseases: From molecular mechanism to therapeutic targets. Signal Transduct. Target. Ther..

[19] Bakhshandeh B., Sorboni S.G., Ranjbar N., Deyhimfar R., Abtahi M.S., Izady M., Kazemi N., Noori A., Pennisi C.P. (2023). Mechanotransduction in tissue engineering: Insights into the interaction of stem cells with biomechanical cues. Exp. Cell Res..

[20] Hsia C.R., Melters D.P., Dalal Y. (2023). The Force is Strong with This Epigenome: Chromatin Structure and Mechanobiology. J. Mol. Biol..

[21] LaBelle J., Wyatt T., Woo S. (2023). Endodermal cells use contact inhibition of locomotion to achieve uniform cell dispersal during zebrafish gastrulation. bioRxiv.

[22] Roycroft A., Mayor R. (2016). Molecular basis of contact inhibition of locomotion. Cell Mol. Life Sci..

[23] Yoon J., Hwang Y.S., Lee M., Sun J., Cho H.J., Knapik L., Daar I.O. (2018). TBC1d24-ephrinB2 interaction regulates contact inhibition of locomotion in neural crest cell migration. Nat. Commun..

[24] Brayford S., Kenny F.N., Hiratsuka T., Serna-Morales E., Yolland L., Luchici A., Stramer B.M. (2019). Heterotypic contact inhibition of locomotion can drive cell sorting between epithelial and mesenchymal cell populations. J. Cell Sci..

[25] Ichikawa T., Stuckenholz C., Davidson L.A. (2020). Non-junctional role of Cadherin3 in cell migration and contact inhibition of locomotion via domain-dependent, opposing regulation of Rac1. Sci. Rep..

[26] Grund A., Till K., Giehl K., Borchers A. (2021). Ptk7 Is Dynamically Localized at Neural Crest Cell-Cell Contact Sites and Functions in Contact Inhibition of Locomotion. Int. J. Mol. Sci..

[27] Kummer D., Steinbacher T., Tholmann S., Schwietzer M.F., Hartmann C., Horenkamp S., Demuth S., Peddibhotla S.S.D., Brinkmann F., Kemper B. (2022). A JAM-A-tetraspanin-alphavbeta5 integrin complex regulates contact inhibition of locomotion. J. Cell Biol..

[28] Noordstra I., Hermoso M.D., Schimmel L., Bonfim-Melo A., Currin-Ross D., Duong C.N., Kalappurakkal J.M., Morris R.G., Vestweber D., Mayor S. (2023). An E-cadherin-actin clutch translates the mechanical force of cortical flow for cell-cell contact to inhibit epithelial cell locomotion. Dev. Cell.

[29] Jain S., Cachoux V.M.L., Narayana G., de Beco S., D’Alessandro J., Cellerin V., Chen T., Heuze M.L., Marcq P., Mege R.M. (2020). The role of single cell mechanical behavior and polarity in driving collective cell migration. Nat. Phys..

[30] Zadeh P., Camley B.A. (2022). Picking winners in cell-cell collisions: Wetting, speed, and contact. Phys. Rev. E.

[31] Garcin C., Straube A. (2019). Microtubules in cell migration. Essays Biochem..

[32] Gao J., Nakamura F. (2022). Actin-Associated Proteins and Small Molecules Targeting the Actin Cytoskeleton. Int. J. Mol. Sci..

[33] Peng N., Nakamura F. (2023). Microtubule-associated proteins and enzymes modifying tubulin. Cytoskeleton.

[34] Bohnet S., Ananthakrishnan R., Mogilner A., Meister J.J., Verkhovsky A.B. (2006). Weak force stalls protrusion at the leading edge of the lamellipodium. Biophys. J..

[35] Pontes B., Monzo P., Gole L., Le Roux A.L., Kosmalska A.J., Tam Z.Y., Luo W., Kan S., Viasnoff V., Roca-Cusachs P. (2017). Membrane tension controls adhesion positioning at the leading edge of cells. J. Cell Biol..

[36] Szabo A., Mayor R. (2018). Mechanisms of Neural Crest Migration. Annu. Rev. Genet..

[37] Bischoff M.C., Lieb S., Renkawitz-Pohl R., Bogdan S. (2021). Filopodia-based contact stimulation of cell migration drives tissue morphogenesis. Nat. Commun..

[38] Singh J., Pagulayan A., Camley B.A., Nain A.S. (2021). Rules of contact inhibition of locomotion for cells on suspended nanofibers. Proc. Natl. Acad. Sci. USA.

[39] Hayakawa M., Hiraiwa T., Wada Y., Kuwayama H., Shibata T. (2020). Polar pattern formation induced by contact following locomotion in a multicellular system. Elife.

[40] Li D., Wang Y.L. (2018). Coordination of cell migration mediated by site-dependent cell-cell contact. Proc. Natl. Acad. Sci. USA.

[41] Pajic-Lijakovic I., Milivojevic M. (2023). Cell jamming-to-unjamming transitions and vice versa in development: Physical aspects. Biosystems.

[42] Malinverno C., Corallino S., Giavazzi F., Bergert M., Li Q., Leoni M., Disanza A., Frittoli E., Oldani A., Martini E. (2017). Endocytic reawakening of motility in jammed epithelia. Nat. Mater..

[43] Palamidessi A., Malinverno C., Frittoli E., Corallino S., Barbieri E., Sigismund S., Beznoussenko G.V., Martini E., Garre M., Ferrara I. (2019). Unjamming overcomes kinetic and proliferation arrest in terminally differentiated cells and promotes collective motility of carcinoma. Nat. Mater..

[44] Molinie N., Rubtsova S.N., Fokin A., Visweshwaran S.P., Rocques N., Polesskaya A., Schnitzler A., Vacher S., Denisov E.V., Tashireva L.A. (2019). Cortical branched actin determines cell cycle progression. Cell Res..

[45] van Helvert S., Storm C., Friedl P. (2018). Mechanoreciprocity in cell migration. Nat. Cell Biol..

[46] Piccolo S., Panciera T., Contessotto P., Cordenonsi M. (2023). YAP/TAZ as master regulators in cancer: Modulation, function and therapeutic approaches. Nat. Cancer.

[47] Guo Y., Luo J., Zou H., Liu C., Deng L., Li P. (2022). Context-dependent transcriptional regulations of YAP/TAZ in cancer. Cancer Lett..

[48] Wehling L., Keegan L., Fernandez-Palanca P., Hassan R., Ghallab A., Schmitt J., Tang Y., Le Marois M., Roessler S., Schirmacher P. (2022). Spatial modeling reveals nuclear phosphorylation and subcellular shuttling of YAP upon drug-induced liver injury. Elife.

[49] McClatchey A.I., Yap A.S. (2012). Contact inhibition (of proliferation) redux. Curr. Opin. Cell Biol..

[50] Fan R., Kim N.G., Gumbiner B.M. (2013). Regulation of Hippo pathway by mitogenic growth factors via phosphoinositide 3-kinase and phosphoinositide-dependent kinase-1. Proc. Natl. Acad. Sci. USA.

[51] Mendonsa A.M., Na T.Y., Gumbiner B.M. (2018). E-cadherin in contact inhibition and cancer. Oncogene.

[52] Senju Y., Hibino E. (2023). Moesin-ezrin-radixin-like protein merlin: Its conserved and distinct functions from those of ERM proteins. Biochim. Biophys. Acta Biomembr..

[53] Kim J.H., Kushiro K., Graham N.A., Asthagiri A.R. (2009). Tunable interplay between epidermal growth factor and cell-cell contact governs the spatial dynamics of epithelial growth. Proc. Natl. Acad. Sci. USA.

[54] Ozawa M. (2015). The N-cadherin cytoplasmic domain confers anchorage-independent growth and the loss of contact inhibition. Sci. Rep..

[55] Chen Q., Zhang N., Xie R., Wang W., Cai J., Choi K.S., David K.K., Huang B., Yabuta N., Nojima H. (2015). Homeostatic control of Hippo signaling activity revealed by an endogenous activating mutation in YAP. Genes. Dev..

[56] Franklin J.M., Ghosh R.P., Shi Q., Reddick M.P., Liphardt J.T. (2020). Concerted localization-resets precede YAP-dependent transcription. Nat. Commun..

[57] Ippolito F., Consalvi V., Noce V., Battistelli C., Cicchini C., Tripodi M., Amicone L., Marchetti A. (2023). Extracellular signal-Regulated Kinase 5 (ERK5) is required for the Yes-associated protein (YAP) co-transcriptional activity. Cell Death Dis..

[58] Yamana S., Tokiyama A., Mizutani K., Hirata K., Takai Y., Rikitake Y. (2015). The Cell Adhesion Molecule Necl-4/CADM4 Serves as a Novel Regulator for Contact Inhibition of Cell Movement and Proliferation. PLoS ONE.

[59] Fan S., Smith M.S., Keeney J., O’Leary M.N., Nusrat A., Parkos C.A. (2022). JAM-A signals through the Hippo pathway to regulate intestinal epithelial proliferation. iScience.

[60] Dupont S., Morsut L., Aragona M., Enzo E., Giulitti S., Cordenonsi M., Zanconato F., Le Digabel J., Forcato M., Bicciato S. (2011). Role of YAP/TAZ in mechanotransduction. Nature.

[61] Aragona M., Panciera T., Manfrin A., Giulitti S., Michielin F., Elvassore N., Dupont S., Piccolo S. (2013). A mechanical checkpoint controls multicellular growth through YAP/TAZ regulation by actin-processing factors. Cell.

[62] Elosegui-Artola A., Andreu I., Beedle A.E.M., Lezamiz A., Uroz M., Kosmalska A.J., Oria R., Kechagia J.Z., Rico-Lastres P., Le Roux A.L. (2017). Force Triggers YAP Nuclear Entry by Regulating Transport across Nuclear Pores. Cell.

[63] Garcia-Garcia M., Sanchez-Perales S., Jarabo P., Calvo E., Huyton T., Fu L., Ng S.C., Sotodosos-Alonso L., Vazquez J., Casas-Tinto S. (2022). Mechanical control of nuclear import by Importin-7 is regulated by its dominant cargo YAP. Nat. Commun..

[64] Elbediwy A., Vanyai H., Diaz-de-la-Loza M.D., Frith D., Snijders A.P., Thompson B.J. (2018). Enigma proteins regulate YAP mechanotransduction. J. Cell Sci..

[65] Sugihara T., Werneburg N.W., Hernandez M.C., Yang L., Kabashima A., Hirsova P., Yohanathan L., Sosa C., Truty M.J., Vasmatzis G. (2018). YAP Tyrosine Phosphorylation and Nuclear Localization in Cholangiocarcinoma Cells Are Regulated by LCK and Independent of LATS Activity. Mol. Cancer Res..

[66] Sidor C., Borreguero-Munoz N., Fletcher G.C., Elbediwy A., Guillermin O., Thompson B.J. (2019). Mask family proteins ANKHD1 and ANKRD17 regulate YAP nuclear import and stability. Elife.

[67] Fletcher G.C., Diaz-de-la-Loza M.D., Borreguero-Munoz N., Holder M., Aguilar-Aragon M., Thompson B.J. (2018). Mechanical strain regulates the Hippo pathway in Drosophila. Development.

[68] Cho Y.S., Jiang J. (2021). Hippo-Independent Regulation of Yki/Yap/Taz: A Non-canonical View. Front. Cell Dev. Biol..

[69] Amirifar P., Kissil J. (2023). The role of Motin family proteins in tumorigenesis-an update. Oncogene.

[70] Chan S.W., Lim C.J., Guo F., Tan I., Leung T., Hong W. (2013). Actin-binding and cell proliferation activities of angiomotin family members are regulated by Hippo pathway-mediated phosphorylation. J. Biol. Chem..

[71] Devany J., Falk M.J., Holt L.J., Murugan A., Gardel M.L. (2023). Epithelial tissue confinement inhibits cell growth and leads to volume-reducing divisions. Dev. Cell.

[72] Karska J., Kowalski S., Saczko J., Moisescu M.G., Kulbacka J. (2023). Mechanosensitive Ion Channels and Their Role in Cancer Cells. Membranes.

[73] Pagnozzi L.A., Butcher J.T. (2017). Mechanotransduction Mechanisms in Mitral Valve Physiology and Disease Pathogenesis. Front. Cardiovasc. Med..

[74] Cheng B., Liu Y., Zhao Y., Li Q., Liu Y., Wang J., Chen Y., Zhang M. (2019). The role of anthrax toxin protein receptor 1 as a new mechanosensor molecule and its mechanotransduction in BMSCs under hydrostatic pressure. Sci. Rep..

[75] He L., Tao J., Maity D., Si F., Wu Y., Wu T., Prasath V., Wirtz D., Sun S.X. (2018). Role of membrane-tension gated Ca^2+^ flux in cell mechanosensation. J. Cell Sci..

[76] Allen A., Maddala R., Eldawy C., Rao P.V. (2022). Mechanical Load and Piezo1 Channel Regulated Myosin II Activity in Mouse Lenses. Int. J. Mol. Sci..

[77] Ellefsen K.L., Holt J.R., Chang A.C., Nourse J.L., Arulmoli J., Mekhdjian A.H., Abuwarda H., Tombola F., Flanagan L.A., Dunn A.R. (2019). Myosin-II mediated traction forces evoke localized Piezo1-dependent Ca^2+^ flickers. Commun. Biol..

[78] Wang M., Ivanovska I., Vashisth M., Discher D.E. (2022). Nuclear mechanoprotection: From tissue atlases as blueprints to distinctive regulation of nuclear lamins. APL Bioeng..

[79] Acheta J., Bhatia U., Haley J., Hong J., Rich K., Close R., Bechler M.E., Belin S., Poitelon Y. (2022). Piezo channels contribute to the regulation of myelination in Schwann cells. Glia.

[80] Puri B.K. (2020). Calcium Signaling and Gene Expression. Adv. Exp. Med. Biol..

[81] Sours-Brothers S., Ma R., Koulen P. (2009). Ca^2+^-sensitive transcriptional regulation: Direct DNA interaction by DREAM. Front. Biosci..

[82] Zeng Y. (2017). Endothelial glycocalyx as a critical signalling platform integrating the extracellular haemodynamic forces and chemical signalling. J. Cell Mol. Med..

[83] Bastianello G., Foiani M. (2023). Mechanisms controlling the mechanical properties of the nuclei. Curr. Opin. Cell Biol..

[84] Maurer M., Lammerding J. (2019). The Driving Force: Nuclear Mechanotransduction in Cellular Function, Fate, and Disease. Annu. Rev. Biomed. Eng..

[85] Phuyal S., Romani P., Dupont S., Farhan H. (2023). Mechanobiology of organelles: Illuminating their roles in mechanosensing and mechanotransduction. Trends Cell Biol..

[86] Wada K., Itoga K., Okano T., Yonemura S., Sasaki H. (2011). Hippo pathway regulation by cell morphology and stress fibers. Development.

[87] Feng M., Wang J., Li K., Nakamura F. (2023). UBE2A/B is the trans-acting factor mediating mechanotransduction and contact inhibition. Biochem. J..

[88] Witke W., Sharpe A.H., Hartwig J.H., Azuma T., Stossel T.P., Kwiatkowski D.J. (1995). Hemostatic, inflammatory, and fibroblast responses are blunted in mice lacking gelsolin. Cell.

[89] Footer M.J., Kerssemakers J.W., Theriot J.A., Dogterom M. (2007). Direct measurement of force generation by actin filament polymerization using an optical trap. Proc. Natl. Acad. Sci. USA.

[90] Kolomeisky A.B., Fisher M.E. (2001). Force-velocity relation for growing microtubules. Biophys. J..

[91] Dogterom M., Yurke B. (1997). Measurement of the force-velocity relation for growing microtubules. Science.

[92] Finer J.T., Simmons R.M., Spudich J.A. (1994). Single myosin molecule mechanics: Piconewton forces and nanometre steps. Nature.

[93] Okada Y., Higuchi H., Hirokawa N. (2003). Processivity of the single-headed kinesin KIF1A through biased binding to tubulin. Nature.

[94] Gennerich A., Carter A.P., Reck-Peterson S.L., Vale R.D. (2007). Force-induced bidirectional stepping of cytoplasmic dynein. Cell.

[95] Sharma S., Subramani S., Popa I. (2021). Does protein unfolding play a functional role in vivo?. FEBS J..

[96] del Rio A., Perez-Jimenez R., Liu R., Roca-Cusachs P., Fernandez J.M., Sheetz M.P. (2009). Stretching single talin rod molecules activates vinculin binding. Science.

[97] Wang Y., Yao M., Baker K.B., Gough R.E., Le S., Goult B.T., Yan J. (2021). Force-Dependent Interactions between Talin and Full-Length Vinculin. J. Am. Chem. Soc..

[98] Goult B.T., Brown N.H., Schwartz M.A. (2021). Talin in mechanotransduction and mechanomemory at a glance. J. Cell Sci..

[99] Nakamura F., Stossel T.P., Hartwig J.H. (2011). The filamins: Organizers of cell structure and function. Cell Adh. Migr..

[100] Ehrlicher A.J., Nakamura F., Hartwig J.H., Weitz D.A., Stossel T.P. (2011). Mechanical strain in actin networks regulates FilGAP and integrin binding to filamin A. Nature.

[101] Grashoff C., Hoffman B.D., Brenner M.D., Zhou R., Parsons M., Yang M.T., McLean M.A., Sligar S.G., Chen C.S., Ha T. (2010). Measuring mechanical tension across vinculin reveals regulation of focal adhesion dynamics. Nature.

[102] Yonemura S., Wada Y., Watanabe T., Nagafuchi A., Shibata M. (2010). alpha-Catenin as a tension transducer that induces adherens junction development. Nat. Cell Biol..

[103] Yao M., Qiu W., Liu R., Efremov A.K., Cong P., Seddiki R., Payre M., Lim C.T., Ladoux B., Mege R.M. (2014). Force-dependent conformational switch of alpha-catenin controls vinculin binding. Nat. Commun..

[104] Le S., Hu X., Yao M., Chen H., Yu M., Xu X., Nakazawa N., Margadant F.M., Sheetz M.P., Yan J. (2017). Mechanotransmission and Mechanosensing of Human alpha-Actinin 1. Cell Rep..

[105] Wallace M., Fedorchak G.R., Agrawal R., Gilbert R.M., Patel J., Park S., Paszek M., Lammerding J. (2023). The lamin A/C Ig-fold undergoes cell density-dependent changes that alter epitope binding. Nucleus.

[106] Zhang X., Halvorsen K., Zhang C.Z., Wong W.P., Springer T.A. (2009). Mechanoenzymatic cleavage of the ultralarge vascular protein von Willebrand factor. Science.

[107] Arce N.A., Cao W., Brown A.K., Legan E.R., Wilson M.S., Xu E.R., Berndt M.C., Emsley J., Zhang X.F., Li R. (2021). Activation of von Willebrand factor via mechanical unfolding of its discontinuous autoinhibitory module. Nat. Commun..

[108] Baneyx G., Baugh L., Vogel V. (2002). Fibronectin extension and unfolding within cell matrix fibrils controlled by cytoskeletal tension. Proc. Natl. Acad. Sci. USA.

[109] Klotzsch E., Smith M.L., Kubow K.E., Muntwyler S., Little W.C., Beyeler F., Gourdon D., Nelson B.J., Vogel V. (2009). Fibronectin forms the most extensible biological fibers displaying switchable force-exposed cryptic binding sites. Proc. Natl. Acad. Sci. USA.

[110] Cao L., Nicosia J., Larouche J., Zhang Y., Bachman H., Brown A.C., Holmgren L., Barker T.H. (2017). Detection of an Integrin-Binding Mechanoswitch within Fibronectin during Tissue Formation and Fibrosis. ACS Nano.

[111] Sawada Y., Tamada M., Dubin-Thaler B.J., Cherniavskaya O., Sakai R., Tanaka S., Sheetz M.P. (2006). Force sensing by mechanical extension of the Src family kinase substrate p130Cas. Cell.

[112] Branis J., Pataki C., Sporrer M., Gerum R.C., Mainka A., Cermak V., Goldmann W.H., Fabry B., Brabek J., Rosel D. (2017). The role of focal adhesion anchoring domains of CAS in mechanotransduction. Sci. Rep..

[113] Bauer M.S., Baumann F., Daday C., Redondo P., Durner E., Jobst M.A., Milles L.F., Mercadante D., Pippig D.A., Gaub H.E. (2019). Structural and mechanistic insights into mechanoactivation of focal adhesion kinase. Proc. Natl. Acad. Sci. USA.

[114] Bazellieres E., Le Bivic A. (2021). Mechanoregulation of PDZ Proteins, An Emerging Function. Methods Mol. Biol..

[115] Dembo M., Torney D.C., Saxman K., Hammer D. (1988). The reaction-limited kinetics of membrane-to-surface adhesion and detachment. Proc. R. Soc. Lond. B Biol. Sci..

[116] Kong F., Garcia A.J., Mould A.P., Humphries M.J., Zhu C. (2009). Demonstration of catch bonds between an integrin and its ligand. J. Cell Biol..

[117] Morikis V.A., Chase S., Wun T., Chaikof E.L., Magnani J.L., Simon S.I. (2017). Selectin catch-bonds mechanotransduce integrin activation and neutrophil arrest on inflamed endothelium under shear flow. Blood.

[118] Yago T., Wu J., Wey C.D., Klopocki A.G., Zhu C., McEver R.P. (2004). Catch bonds govern adhesion through L-selectin at threshold shear. J. Cell Biol..

[119] Liu B., Chen W., Evavold B.D., Zhu C. (2014). Accumulation of dynamic catch bonds between TCR and agonist peptide-MHC triggers T cell signaling. Cell.

[120] Zhu C., Chen W., Lou J., Rittase W., Li K. (2019). Mechanosensing through immunoreceptors. Nat. Immunol..

[121] Pettmann J., Awada L., Rozycki B., Huhn A., Faour S., Kutuzov M., Limozin L., Weikl T.R., van der Merwe P.A., Robert P. (2023). Mechanical forces impair antigen discrimination by reducing differences in T-cell receptor/peptide-MHC off-rates. EMBO J..

[122] Chen W., Lou J., Zhu C. (2010). Forcing switch from short- to intermediate- and long-lived states of the alphaA domain generates LFA-1/ICAM-1 catch bonds. J. Biol. Chem..

[123] Buckley C.D., Tan J., Anderson K.L., Hanein D., Volkmann N., Weis W.I., Nelson W.J., Dunn A.R. (2014). Cell adhesion. The minimal cadherin-catenin complex binds to actin filaments under force. Science.

[124] Fang L., Zhao Y., Guo P., Fang Y., Wu J. (2023). MD Simulation Reveals Regulation of Mechanical Force and Extracellular Domain 2 on Binding of DNAM-1 to CD155. Molecules.

[125] Zhao X., Kolawole E.M., Chan W., Feng Y., Yang X., Gee M.H., Jude K.M., Sibener L.V., Fordyce P.M., Germain R.N. (2022). Tuning T cell receptor sensitivity through catch bond engineering. Science.

[126] Puchner E.M., Alexandrovich A., Kho A.L., Hensen U., Schafer L.V., Brandmeier B., Grater F., Grubmuller H., Gaub H.E., Gautel M. (2008). Mechanoenzymatics of titin kinase. Proc. Natl. Acad. Sci. USA.

[127] Lim C.G., Jang J., Kim C. (2018). Cellular machinery for sensing mechanical force. BMB Rep..

[128] Chuang Y.C., Chen C.C. (2022). Force From Filaments: The Role of the Cytoskeleton and Extracellular Matrix in the Gating of Mechanosensitive Channels. Front. Cell Dev. Biol..

[129] Lin Y.C., Guo Y.R., Miyagi A., Levring J., MacKinnon R., Scheuring S. (2019). Force-induced conformational changes in PIEZO1. Nature.

[130] Liu C., Montell C. (2015). Forcing open TRP channels: Mechanical gating as a unifying activation mechanism. Biochem. Biophys. Res. Commun..

[131] Liu Y.S., Liu Y.A., Huang C.J., Yen M.H., Tseng C.T., Chien S., Lee O.K. (2015). Mechanosensitive TRPM7 mediates shear stress and modulates osteogenic differentiation of mesenchymal stromal cells through Osterix pathway. Sci. Rep..

[132] Starostina I., Jang Y.K., Kim H.S., Suh J.S., Ahn S.H., Choi G.H., Suk M., Kim T.J. (2021). Distinct calcium regulation of TRPM7 mechanosensitive channels at plasma membrane microdomains visualized by FRET-based single cell imaging. Sci. Rep..

[133] Maingret F., Fosset M., Lesage F., Lazdunski M., Honore E. (1999). TRAAK is a mammalian neuronal mechano-gated K+ channel. J. Biol. Chem..

[134] Lesage F., Terrenoire C., Romey G., Lazdunski M. (2000). Human TREK2, a 2P domain mechano-sensitive K+ channel with multiple regulations by polyunsaturated fatty acids, lysophospholipids, and Gs, Gi, and Gq protein-coupled receptors. J. Biol. Chem..

[135] Herrera-Perez S., Lamas J.A. (2023). TREK channels in Mechanotransduction: A Focus on the Cardiovascular System. Front. Cardiovasc. Med..

[136] Yang H., Zhang G., Cui J. (2015). BK channels: Multiple sensors, one activation gate. Front. Physiol..

[137] Murthy S.E., Dubin A.E., Whitwam T., Jojoa-Cruz S., Cahalan S.M., Mousavi S.A.R., Ward A.B., Patapoutian A. (2018). OSCA/TMEM63 are an Evolutionarily Conserved Family of Mechanically Activated Ion Channels. Elife.

[138] Bianchi L. (2021). DEG/ENaC Ion Channels in the Function of the Nervous System: From Worm to Man. Adv. Exp. Med. Biol..

[139] Dong X., Zhao B., Iacob R.E., Zhu J., Koksal A.C., Lu C., Engen J.R., Springer T.A. (2017). Force interacts with macromolecular structure in activation of TGF-beta. Nature.

[140] Kossatz S., Beer A.J., Notni J. (2021). It’s Time to Shift the Paradigm: Translation and Clinical Application of Non-alphavbeta3 Integrin Targeting Radiopharmaceuticals. Cancers.

[141] Rognoni L., Stigler J., Pelz B., Ylanne J., Rief M. (2012). Dynamic force sensing of filamin revealed in single-molecule experiments. Proc. Natl. Acad. Sci. USA.

[142] Tsuda Y., Yasutake H., Ishijima A., Yanagida T. (1996). Torsional rigidity of single actin filaments and actin-actin bond breaking force under torsion measured directly by in vitro micromanipulation. Proc. Natl. Acad. Sci. USA.

[143] Endow S.A., Marszalek P.E. (2019). An estimate to the first approximation of microtubule rupture force. Eur. Biophys. J..

[144] Raffa V. (2023). Force: A messenger of axon outgrowth. Semin. Cell Dev. Biol..

[145] Chaudhuri O., Parekh S.H., Fletcher D.A. (2007). Reversible stress softening of actin networks. Nature.

[146] Liu C., Gao X., Li Y., Sun W., Xu Y., Tan Y., Du R., Zhong G., Zhao D., Liu Z. (2022). The mechanosensitive lncRNA Neat1 promotes osteoblast function through paraspeckle-dependent Smurf1 mRNA retention. Bone Res..

[147] Kassianidou E., Kalita J., Lim R.Y.H. (2019). The role of nucleocytoplasmic transport in mechanotransduction. Exp. Cell Res..

[148] Puliafito A., Hufnagel L., Neveu P., Streichan S., Sigal A., Fygenson D.K., Shraiman B.I. (2012). Collective and single cell behavior in epithelial contact inhibition. Proc. Natl. Acad. Sci. USA.

[149] Streichan S.J., Hoerner C.R., Schneidt T., Holzer D., Hufnagel L. (2014). Spatial constraints control cell proliferation in tissues. Proc. Natl. Acad. Sci. USA.

[150] Vanmunster M., Rojo-Garcia A.V., Pacolet A., Jonkers I., Koppo K., Lories R., Suhr F. (2023). Prolonged mechanical muscle loading increases mechanosensor gene and protein levels and causes a moderate fast-to-slow fiber type switch in mice. J. Appl. Physiol..

[151] Swift J., Ivanovska I.L., Buxboim A., Harada T., Dingal P.C., Pinter J., Pajerowski J.D., Spinler K.R., Shin J.W., Tewari M. (2013). Nuclear lamin-A scales with tissue stiffness and enhances matrix-directed differentiation. Science.

[152] Urciuoli E., Peruzzi B. (2022). The Paradox of Nuclear Lamins in Pathologies: Apparently Controversial Roles Explained by Tissue-Specific Mechanobiology. Cells.

[153] Owens D.J., Fischer M., Jabre S., Moog S., Mamchaoui K., Butler-Browne G., Coirault C. (2020). Lamin Mutations Cause Increased YAP Nuclear Entry in Muscle Stem Cells. Cells.

[154] Dubik N., Mai S. (2020). Lamin A/C: Function in Normal and Tumor Cells. Cancers.

[155] Santini G.T., Shah P.P., Karnay A., Jain R. (2022). Aberrant chromatin organization at the nexus of laminopathy disease pathways. Nucleus.

[156] Guelen L., Pagie L., Brasset E., Meuleman W., Faza M.B., Talhout W., Eussen B.H., de Klein A., Wessels L., de Laat W. (2008). Domain organization of human chromosomes revealed by mapping of nuclear lamina interactions. Nature.

[157] Lityagina O., Dobreva G. (2021). The LINC Between Mechanical Forces and Chromatin. Front. Physiol..

[158] Takata T., Matsumura M. (2022). The LINC Complex Assists the Nuclear Import of Mechanosensitive Transcriptional Regulators. Results Probl. Cell Differ..

[159] Cho S., Irianto J., Discher D.E. (2017). Mechanosensing by the nucleus: From pathways to scaling relationships. J. Cell Biol..

[160] Patil S., Deshpande S., Sengupta K. (2023). Nuclear envelope protein lamin B receptor protects the genome from chromosomal instability and tumorigenesis. Hum. Mol. Genet..

[161] Liddane A.G., Holaska J.M. (2021). The Role of Emerin in Cancer Progression and Metastasis. Int. J. Mol. Sci..

[162] Lammerding J., Hsiao J., Schulze P.C., Kozlov S., Stewart C.L., Lee R.T. (2005). Abnormal nuclear shape and impaired mechanotransduction in emerin-deficient cells. J. Cell Biol..

[163] Manzo S.G., Dauban L., van Steensel B. (2022). Lamina-associated domains: Tethers and looseners. Curr. Opin. Cell Biol..

[164] Kiseleva A.A., Cheng Y.C., Smith C.L., Katz R.A., Poleshko A. (2023). PRR14 organizes H3K9me3-modified heterochromatin at the nuclear lamina. Nucleus.

[165] Taniura H., Glass C., Gerace L. (1995). A chromatin binding site in the tail domain of nuclear lamins that interacts with core histones. J. Cell Biol..

[166] Liu S., Li Y., Hong Y., Wang M., Zhang H., Ma J., Qu K., Huang G., Lu T.J. (2023). Mechanotherapy in oncology: Targeting nuclear mechanics and mechanotransduction. Adv. Drug Deliv. Rev..

[167] Almonacid M., Terret M.E., Verlhac M.H. (2019). Nuclear positioning as an integrator of cell fate. Curr. Opin. Cell Biol..

[168] Lombardi M.L., Jaalouk D.E., Shanahan C.M., Burke B., Roux K.J., Lammerding J. (2011). The interaction between nesprins and sun proteins at the nuclear envelope is critical for force transmission between the nucleus and cytoskeleton. J. Biol. Chem..

[169] Thiam H.R., Vargas P., Carpi N., Crespo C.L., Raab M., Terriac E., King M.C., Jacobelli J., Alberts A.S., Stradal T. (2016). Perinuclear Arp2/3-driven actin polymerization enables nuclear deformation to facilitate cell migration through complex environments. Nat. Commun..

[170] Korfali N., Florens L., Schirmer E.C. (2016). Isolation, Proteomic Analysis, and Microscopy Confirmation of the Liver Nuclear Envelope Proteome. Methods Mol. Biol..

[171] Li K., Li Y., Nakamura F. (2023). Identification and partial characterization of new cell density-dependent nucleocytoplasmic shuttling proteins and open chromatin. Sci. Rep..

[172] Agelopoulos M., Foutadakis S., Thanos D. (2021). The Causes and Consequences of Spatial Organization of the Genome in Regulation of Gene Expression. Front. Immunol..

[173] Park S., Kim G.W., Kwon S.H., Lee J.S. (2020). Broad domains of histone H3 lysine 4 trimethylation in transcriptional regulation and disease. FEBS J..

[174] Wang H., Fan Z., Shliaha P.V., Miele M., Hendrickson R.C., Jiang X., Helin K. (2023). H3K4me3 regulates RNA polymerase II promoter-proximal pause-release. Nature.

[175] Lin J., Wu Y., Tian G., Yu D., Yang E., Lam W.H., Liu Z., Jing Y., Dang S., Bao X. (2023). Menin “reads” H3K79me2 mark in a nucleosomal context. Science.

[176] Boeri L., Albani D., Raimondi M.T., Jacchetti E. (2019). Mechanical regulation of nucleocytoplasmic translocation in mesenchymal stem cells: Characterization and methods for investigation. Biophys. Rev..

[177] Pocaterra A., Romani P., Dupont S. (2020). YAP/TAZ functions and their regulation at a glance. J. Cell Sci..

[178] Koushki N., Ghagre A., Srivastava L.K., Molter C., Ehrlicher A.J. (2023). Nuclear compression regulates YAP spatiotemporal fluctuations in living cells. Proc. Natl. Acad. Sci. USA.

[179] Li Y., Li S., Wu H. (2022). Ubiquitination-Proteasome System (UPS) and Autophagy Two Main Protein Degradation Machineries in Response to Cell Stress. Cells.

[180] Kolobynina K.G., Rapp A., Cardoso M.C. (2022). Chromatin Ubiquitination Guides DNA Double Strand Break Signaling and Repair. Front. Cell Dev. Biol..

[181] Sekiguchi M., Matsushita N. (2022). DNA Damage Response Regulation by Histone Ubiquitination. Int. J. Mol. Sci..

[182] Wang J., Zhou Q., Ding J., Yin T., Ye P., Zhang Y. (2022). The Conceivable Functions of Protein Ubiquitination and Deubiquitination in Reproduction. Front. Physiol..

[183] Sengupta M., Pluciennik A., Merry D.E. (2022). The role of ubiquitination in spinal and bulbar muscular atrophy. Front. Mol. Neurosci..

[184] Zhong T., Lei K., Lin X., Xie Z., Luo S., Zhou Z., Zhao B., Li X. (2022). Protein ubiquitination in T cell development. Front. Immunol..

[185] Johnston R.K., Balasubramanian S., Kasiganesan H., Baicu C.F., Zile M.R., Kuppuswamy D. (2009). Beta3 integrin-mediated ubiquitination activates survival signaling during myocardial hypertrophy. FASEB J..

[186] Jiang X., Austin P.F., Niederhoff R.A., Manson S.R., Riehm J.J., Cook B.L., Pengue G., Chitaley K., Nakayama K., Nakayama K.I. (2009). Mechanoregulation of proliferation. Mol. Cell Biol..

[187] Ulbricht A., Eppler F.J., Tapia V.E., van der Ven P.F., Hampe N., Hersch N., Vakeel P., Stadel D., Haas A., Saftig P. (2013). Cellular mechanotransduction relies on tension-induced and chaperone-assisted autophagy. Curr. Biol..

[188] Yang N., Chen T., Wang L., Liu R., Niu Y., Sun L., Yao B., Wang Y., Yang W., Liu Q. (2020). CXCR4 mediates matrix stiffness-induced downregulation of UBTD1 driving hepatocellular carcinoma progression via YAP signaling pathway. Theranostics.

[189] Ji C., Zhang J., Zhu Y., Shi H., Yin S., Sun F., Wang Q., Zhang L., Yan Y., Zhang X. (2020). Exosomes derived from hucMSC attenuate renal fibrosis through CK1delta/beta-TRCP-mediated YAP degradation. Cell Death Dis..

[190] Liu H., Zhong L., Lu Y., Liu X., Wei J., Ding Y., Huang H., Nie Q., Liao X. (2022). Deubiquitylase OTUD1 confers Erlotinib sensitivity in non-small cell lung cancer through inhibition of nuclear translocation of YAP1. Cell Death Discov..

[191] Li R., Shao J., Jin Y.J., Kawase H., Ong Y.T., Troidl K., Quan Q., Wang L., Bonnavion R., Wietelmann A. (2023). Endothelial FAT1 inhibits angiogenesis by controlling YAP/TAZ protein degradation via E3 ligase MIB2. Nat. Commun..

[192] Torrino S., Roustan F.R., Kaminski L., Bertero T., Pisano S., Ambrosetti D., Dufies M., Uhler J.P., Lemichez E., Mettouchi A. (2019). UBTD1 is a mechano-regulator controlling cancer aggressiveness. EMBO Rep..

[193] Zhao B., Li L., Tumaneng K., Wang C.Y., Guan K.L. (2010). A coordinated phosphorylation by Lats and CK1 regulates YAP stability through SCF(beta-TRCP). Genes. Dev..

[194] Chitragari G., Shalaby S.Y., Sumpio B.J., Kurita J., Sumpio B.E. (2018). Regulation of Yes-Associated Protein by Laminar Flow. Ann. Vasc. Surg..

[195] Sun X., Ding Y., Zhan M., Li Y., Gao D., Wang G., Gao Y., Li Y., Wu S., Lu L. (2019). Usp7 regulates Hippo pathway through deubiquitinating the transcriptional coactivator Yorkie. Nat. Commun..

[196] Yao F., Zhou Z., Kim J., Hang Q., Xiao Z., Ton B.N., Chang L., Liu N., Zeng L., Wang W. (2018). SKP2- and OTUD1-regulated non-proteolytic ubiquitination of YAP promotes YAP nuclear localization and activity. Nat. Commun..

[197] Osborne H.C., Irving E., Forment J.V., Schmidt C.K. (2021). E2 enzymes in genome stability: Pulling the strings behind the scenes. Trends Cell Biol..

[198] Pavel M., Renna M., Park S.J., Menzies F.M., Ricketts T., Fullgrabe J., Ashkenazi A., Frake R.A., Lombarte A.C., Bento C.F. (2018). Contact inhibition controls cell survival and proliferation via YAP/TAZ-autophagy axis. Nat. Commun..

[199] Sun Y., Wang H., Qu T., Luo J., An P., Ren F., Luo Y., Li Y. (2023). mTORC2: A multifaceted regulator of autophagy. Cell Commun. Signal..

[200] Boutahar N., Guignandon A., Vico L., Lafage-Proust M.H. (2004). Mechanical strain on osteoblasts activates autophosphorylation of focal adhesion kinase and proline-rich tyrosine kinase 2 tyrosine sites involved in ERK activation. J. Biol. Chem..

[201] Hernandez-Caceres M.P., Munoz L., Pradenas J.M., Pena F., Lagos P., Aceiton P., Owen G.I., Morselli E., Criollo A., Ravasio A. (2021). Mechanobiology of Autophagy: The Unexplored Side of Cancer. Front. Oncol..

[202] Ravasio A., Morselli E., Bertocchi C. (2022). Mechanoautophagy: Synergies Between Autophagy and Cell Mechanotransduction at Adhesive Complexes. Front. Cell Dev. Biol..

[203] Mao Z., Nakamura F. (2020). Structure and Function of Filamin C in the Muscle Z-Disc. Int. J. Mol. Sci..

[204] Jain M., Weber A., Maly K., Manjaly G., Deek J., Tsvyetkova O., Stulic M., Toca-Herrera J.L., Jantsch M.F. (2022). A-to-I RNA editing of Filamin A regulates cellular adhesion, migration and mechanical properties. FEBS J..

[205] Sethi R., Seppala J., Tossavainen H., Ylilauri M., Ruskamo S., Pentikainen O.T., Pentikainen U., Permi P., Ylanne J. (2014). A novel structural unit in the N-terminal region of filamins. J. Biol. Chem..

[206] Nakamura F., Pudas R., Heikkinen O., Permi P., Kilpelainen I., Munday A.D., Hartwig J.H., Stossel T.P., Ylanne J. (2006). The structure of the GPIb-filamin A complex. Blood.

[207] Nakamura F., Osborn T.M., Hartemink C.A., Hartwig J.H., Stossel T.P. (2007). Structural basis of filamin A functions. J. Cell Biol..

[208] Tossavainen H., Koskela O., Jiang P., Ylanne J., Campbell I.D., Kilpelainen I., Permi P. (2012). Model of a six immunoglobulin-like domain fragment of filamin A (16-21) built using residual dipolar couplings. J. Am. Chem. Soc..

[209] Ruskamo S., Gilbert R., Hofmann G., Jiang P., Campbell I.D., Ylanne J., Pentikainen U. (2012). The C-terminal rod 2 fragment of filamin A forms a compact structure that can be extended. Biochem. J..

[210] Beedle A.E., Garcia-Manyes S. (2023). The role of single protein elasticity in mechanobiology. Nat. Rev. Mater..

[211] Deng Y., Yan J. (2023). Force-Dependent Structural Changes of Filamin C Rod Domains Regulated by Filamin C Dimer. J. Am. Chem. Soc..

[212] Wang J., Nakamura F. (2019). Identification of Filamin A Mechanobinding Partner II: Fimbacin Is a Novel Actin Cross-Linking and Filamin A Binding Protein. Biochemistry.

[213] Wang L., Nakamura F. (2019). Identification of Filamin A Mechanobinding Partner I: Smoothelin Specifically Interacts with the Filamin A Mechanosensitive Domain 21. Biochemistry.

[214] Feng Z., Mao Z., Yang Z., Liu X., Nakamura F. (2023). The force-dependent filamin A-G3BP1 interaction regulates phase-separated stress granule formation. J. Cell Sci..

[215] Zhang H., Mao Z., Yang Z., Nakamura F. (2023). Identification of Filamin A Mechanobinding Partner III: SAV1 Specifically Interacts with Filamin A Mechanosensitive Domain 21. Biochemistry.

[216] Mao Z., Nakamura F. (2023). Interaction of LARP4 to filamin A mechanosensing domain regulates cell migrations. Front. Cell Dev. Biol..

[217] Ohta Y., Hartwig J.H., Stossel T.P. (2006). FilGAP, a Rho- and ROCK-regulated GAP for Rac binds filamin A to control actin remodelling. Nat. Cell Biol..

[218] Nakamura F., Heikkinen O., Pentikainen O.T., Osborn T.M., Kasza K.E., Weitz D.A., Kupiainen O., Permi P., Kilpelainen I., Ylanne J. (2009). Molecular basis of filamin A-FilGAP interaction and its impairment in congenital disorders associated with filamin A mutations. PLoS ONE.

[219] Saito K., Mori M., Kambara N., Ohta Y. (2021). FilGAP, a GAP protein for Rac, regulates front-rear polarity and tumor cell migration through the ECM. FASEB J..

[220] Schaks M., Giannone G., Rottner K. (2019). Actin dynamics in cell migration. Essays Biochem..

[221] Machacek M., Hodgson L., Welch C., Elliott H., Pertz O., Nalbant P., Abell A., Johnson G.L., Hahn K.M., Danuser G. (2009). Coordination of Rho GTPase activities during cell protrusion. Nature.

[222] Nakamura F. (2013). FilGAP and its close relatives: A mediator of Rho-Rac antagonism that regulates cell morphology and migration. Biochem. J..

[223] Zimmermann J., Camley B.A., Rappel W.J., Levine H. (2016). Contact inhibition of locomotion determines cell-cell and cell-substrate forces in tissues. Proc. Natl. Acad. Sci. USA.

[224] Koehler S., Huber T.B., Denholm B. (2022). A protective role for &lt;i&gt;Drosophila&lt;/i&gt; Filamin in nephrocytes via Yorkie mediated hypertrophy. Life Sci. Alliance.

[225] Zhang H., Yang Z., Nakamura F. (2023). Importance of the filamin A-Sav1 interaction in organ size control: Evidence from transgenic mice. Int. J. Dev. Biol..

[226] Camargo F.D., Gokhale S., Johnnidis J.B., Fu D., Bell G.W., Jaenisch R., Brummelkamp T.R. (2007). YAP1 increases organ size and expands undifferentiated progenitor cells. Curr. Biol..

[227] Dong J., Feldmann G., Huang J., Wu S., Zhang N., Comerford S.A., Gayyed M.F., Anders R.A., Maitra A., Pan D. (2007). Elucidation of a universal size-control mechanism in Drosophila and mammals. Cell.

[228] Lu L., Finegold M.J., Johnson R.L. (2018). Hippo pathway coactivators Yap and Taz are required to coordinate mammalian liver regeneration. Exp. Mol. Med..

[229] Verboven E., Moya I.M., Sansores-Garcia L., Xie J., Hillen H., Kowalczyk W., Vella G., Verhulst S., Castaldo S.A., Alguero-Nadal A. (2021). Regeneration Defects in Yap and Taz Mutant Mouse Livers Are Caused by Bile Duct Disruption and Cholestasis. Gastroenterology.

[230] Russell J.O., Camargo F.D. (2022). Hippo signalling in the liver: Role in development, regeneration and disease. Nat. Rev. Gastroenterol. Hepatol..

[231] Lee J.H., Kim T.S., Yang T.H., Koo B.K., Oh S.P., Lee K.P., Oh H.J., Lee S.H., Kong Y.Y., Kim J.M. (2008). A crucial role of WW45 in developing epithelial tissues in the mouse. EMBO J..

[232] Lee K.P., Lee J.H., Kim T.S., Kim T.H., Park H.D., Byun J.S., Kim M.C., Jeong W.I., Calvisi D.F., Kim J.M. (2010). The Hippo-Salvador pathway restrains hepatic oval cell proliferation, liver size, and liver tumorigenesis. Proc. Natl. Acad. Sci. USA.

[233] Engler A.J., Sen S., Sweeney H.L., Discher D.E. (2006). Matrix elasticity directs stem cell lineage specification. Cell.

[234] Chowdhury F., Huang B., Wang N. (2022). Forces in stem cells and cancer stem cells. Cells Dev..

[235] Donnaloja F., Limonta E., Mancosu C., Morandi F., Boeri L., Albani D., Raimondi M.T. (2023). Unravelling the mechanotransduction pathways in Alzheimer’s disease. J. Biol. Eng..

[236] Perez-Dominguez S., Lopez-Alonso J., Lafont F., Radmacher M. (2023). Comparison of Rheological Properties of Healthy versus Dupuytren Fibroblasts When Treated with a Cell Contraction Inhibitor by Atomic Force Microscope. Int. J. Mol. Sci..

[237] Swaminathan V., Mythreye K., O’Brien E.T., Berchuck A., Blobe G.C., Superfine R. (2011). Mechanical stiffness grades metastatic potential in patient tumor cells and in cancer cell lines. Cancer Res..

[238] Kawano S., Kojima M., Higuchi Y., Sugimoto M., Ikeda K., Sakuyama N., Takahashi S., Hayashi R., Ochiai A., Saito N. (2015). Assessment of elasticity of colorectal cancer tissue, clinical utility, pathological and phenotypical relevance. Cancer Sci..

[239] Panciera T., Citron A., Di Biagio D., Battilana G., Gandin A., Giulitti S., Forcato M., Bicciato S., Panzetta V., Fusco S. (2020). Reprogramming normal cells into tumour precursors requires ECM stiffness and oncogene-mediated changes of cell mechanical properties. Nat. Mater..

[240] Khetan S., Guvendiren M., Legant W.R., Cohen D.M., Chen C.S., Burdick J.A. (2013). Degradation-mediated cellular traction directs stem cell fate in covalently crosslinked three-dimensional hydrogels. Nat. Mater..

[241] Caliari S.R., Vega S.L., Kwon M., Soulas E.M., Burdick J.A. (2016). Dimensionality and spreading influence MSC YAP/TAZ signaling in hydrogel environments. Biomaterials.

[242] Major L.G., Holle A.W., Young J.L., Hepburn M.S., Jeong K., Chin I.L., Sanderson R.W., Jeong J.H., Aman Z.M., Kennedy B.F. (2019). Volume Adaptation Controls Stem Cell Mechanotransduction. ACS Appl. Mater. Interfaces.

[243] Yahalom-Ronen Y., Rajchman D., Sarig R., Geiger B., Tzahor E. (2015). Reduced matrix rigidity promotes neonatal cardiomyocyte dedifferentiation, proliferation and clonal expansion. Elife.

[244] Dubovy P., Bednarova J. (1999). The extracellular matrix of rat pacinian corpuscles: An analysis of its fine structure. Anat. Embryol..

[245] Huang S., Chen C.S., Ingber D.E. (1998). Control of cyclin D1, p27(Kip1), and cell cycle progression in human capillary endothelial cells by cell shape and cytoskeletal tension. Mol. Biol. Cell.

[246] Moreno-Vicente R., Pavon D.M., Martin-Padura I., Catala-Montoro M., Diez-Sanchez A., Quilez-Alvarez A., Lopez J.A., Sanchez-Alvarez M., Vazquez J., Strippoli R. (2018). Caveolin-1 Modulates Mechanotransduction Responses to Substrate Stiffness through Actin-Dependent Control of YAP. Cell Rep..

[247] Seetharaman S., Vianay B., Roca V., Farrugia A.J., De Pascalis C., Boeda B., Dingli F., Loew D., Vassilopoulos S., Bershadsky A. (2022). Microtubules tune mechanosensitive cell responses. Nat. Mater..

[248] Infante E., Etienne-Manneville S. (2022). Intermediate filaments: Integration of cell mechanical properties during migration. Front. Cell Dev. Biol..

[249] Storm C., Pastore J.J., MacKintosh F.C., Lubensky T.C., Janmey P.A. (2005). Nonlinear elasticity in biological gels. Nature.

[250] Janmey P.A., Hvidt S., Lamb J., Stossel T.P. (1990). Resemblance of actin-binding protein/actin gels to covalently crosslinked networks. Nature.

[251] Mora M., Stannard A., Garcia-Manyes S. (2020). The nanomechanics of individual proteins. Chem. Soc. Rev..

[252] Mao Y., Nielsen P., Ali J. (2022). Passive and Active Microrheology for Biomedical Systems. Front. Bioeng. Biotechnol..

[253] Efremov Y.M., Okajima T., Raman A. (2020). Measuring viscoelasticity of soft biological samples using atomic force microscopy. Soft Matter.

[254] Caponi S., Passeri A., Capponi G., Fioretto D., Vassalli M., Mattarelli M. (2022). Non-contact elastography methods in mechanobiology: A point of view. Eur. Biophys. J..

[255] Beedle A.E., Roca-Cusachs P. (2023). The reversibility of cellular mechano-activation. Curr. Opin. Cell Biol..

[256] Choi S.Y., Saravia-Butler A., Shirazi-Fard Y., Leveson-Gower D., Stodieck L.S., Cadena S.M., Beegle J., Solis S., Ronca A., Globus R.K. (2020). Validation of a New Rodent Experimental System to Investigate Consequences of Long Duration Space Habitation. Sci. Rep..

[257] Young K.S., Kim K.H., Rajulu S. (2023). Anthropometric Changes in Spaceflight. Hum. Factors.

[258] Ingber D.E. (2003). Mechanobiology and diseases of mechanotransduction. Ann. Med..

[259] Novak C., Ballinger M.N., Ghadiali S. (2021). Mechanobiology of Pulmonary Diseases: A Review of Engineering Tools to Understand Lung Mechanotransduction. J. Biomech. Eng..

[260] Zuela-Sopilniak N., Lammerding J. (2022). Can’t handle the stress? Mechanobiology and disease. Trends Mol. Med..

[261] Ruffilli A., Viroli G., Neri S., Traversari M., Barile F., Manzetti M., Assirelli E., Ialuna M., Vita F., Faldini C. (2023). Mechanobiology of the Human Intervertebral Disc: Systematic Review of the Literature and Future Perspectives. Int. J. Mol. Sci..

[262] Liu X., Li J., Yue Y., Li J., Wang M., Hao L. (2023). Mechanisms of mechanical force aggravating periodontitis: A review. Oral. Dis..

[263] Park J.S., Burckhardt C.J., Lazcano R., Solis L.M., Isogai T., Li L., Chen C.S., Gao B., Minna J.D., Bachoo R. (2020). Mechanical regulation of glycolysis via cytoskeleton architecture. Nature.

[264] Zanotelli M.R., Zhang J., Reinhart-King C.A. (2021). Mechanoresponsive metabolism in cancer cell migration and metastasis. Cell Metab..

[265] Zanotelli M.R., Zhang J., Ortiz I., Wang W., Chada N.C., Reinhart-King C.A. (2022). Highly motile cells are metabolically responsive to collagen density. Proc. Natl. Acad. Sci. USA.

[266] Zhu W., Chen X., Guo X., Liu H., Ma R., Wang Y., Liang Y., Sun Y., Wang M., Zhao R. (2022). Low Glucose-Induced Overexpression of HOXC-AS3 Promotes Metabolic Reprogramming of Breast Cancer. Cancer Res..

[267] Xie N., Xiao C., Shu Q., Cheng B., Wang Z., Xue R., Wen Z., Wang J., Shi H., Fan D. (2023). Cell response to mechanical microenvironment cues via Rho signaling: From mechanobiology to mechanomedicine. Acta Biomater..

[268] Dawson L.W., Cronin N.M., DeMali K.A. (2023). Mechanotransduction: Forcing a change in metabolism. Curr. Opin. Cell Biol..

[269] Hayward M.K., Muncie J.M., Weaver V.M. (2021). Tissue mechanics in stem cell fate, development, and cancer. Dev. Cell.

[270] Rocha D.N., Carvalho E.D., Relvas J.B., Oliveira M.J., Pego A.P. (2022). Mechanotransduction: Exploring New Therapeutic Avenues in Central Nervous System Pathology. Front. Neurosci..

[271] Nicolas-Boluda A., Vaquero J., Vimeux L., Guilbert T., Barrin S., Kantari-Mimoun C., Ponzo M., Renault G., Deptula P., Pogoda K. (2021). Tumor stiffening reversion through collagen crosslinking inhibition improves T cell migration and anti-PD-1 treatment. Elife.

[272] Raghu G., Brown K.K., Collard H.R., Cottin V., Gibson K.F., Kaner R.J., Lederer D.J., Martinez F.J., Noble P.W., Song J.W. (2017). Efficacy of simtuzumab versus placebo in patients with idiopathic pulmonary fibrosis: A randomised, double-blind, controlled, phase 2 trial. Lancet Respir. Med..

[273] Wang X., Senapati S., Akinbote A., Gnanasambandam B., Park P.S., Senyo S.E. (2020). Microenvironment stiffness requires decellularized cardiac extracellular matrix to promote heart regeneration in the neonatal mouse heart. Acta Biomater..

[274] Ho C.N.Q., Tran M.T., Doan C.C., Hoang S.N., Tran D.H., Le L.T. (2021). Simulated Microgravity Inhibits the Proliferation of Chang Liver Cells by Attenuation of the Major Cell Cycle Regulators and Cytoskeletal Proteins. Int. J. Mol. Sci..

[275] Lawrence H.W. (2023). Human Physiological Limitations to Long-Term Spaceflight and Living in Space. Aerosp. Med. Hum. Perform..

[276] Li W., Shu X., Zhang X., Zhang Z., Sun S., Li N., Long M. (2023). Potential Roles of YAP/TAZ Mechanotransduction in Spaceflight-Induced Liver Dysfunction. Int. J. Mol. Sci..

[277] Liu Y., Chen B.P., Lu M., Zhu Y., Stemerman M.B., Chien S., Shyy J.Y. (2002). Shear stress activation of SREBP1 in endothelial cells is mediated by integrins. Arterioscler. Thromb. Vasc. Biol..

[278] Deschner J., Hofman C.R., Piesco N.P., Agarwal S. (2003). Signal transduction by mechanical strain in chondrocytes. Curr. Opin. Clin. Nutr. Metab. Care.

[279] Chen N.X., Geist D.J., Genetos D.C., Pavalko F.M., Duncan R.L. (2003). Fluid shear-induced NFkappaB translocation in osteoblasts is mediated by intracellular calcium release. Bone.

[280] Young S.R., Gerard-O’Riley R., Harrington M., Pavalko F.M. (2010). Activation of NF-kappaB by fluid shear stress, but not TNF-alpha, requires focal adhesion kinase in osteoblasts. Bone.

[281] Norvell S.M., Alvarez M., Bidwell J.P., Pavalko F.M. (2004). Fluid shear stress induces beta-catenin signaling in osteoblasts. Calcif. Tissue Int..

[282] Yang Z., Bidwell J.P., Young S.R., Gerard-O’Riley R., Wang H., Pavalko F.M. (2010). Nmp4/CIZ inhibits mechanically induced beta-catenin signaling activity in osteoblasts. J. Cell. Physiol..

[283] Liu S., Zhou F., Shen Y., Zhang Y., Yin H., Zeng Y., Liu J., Yan Z., Liu X. (2016). Fluid shear stress induces epithelial-mesenchymal transition (EMT) in Hep-2 cells. Oncotarget.

[284] Li F.F., Zhang B., Cui J.H., Chen F.L., Ding Y., Feng X. (2018). Alterations in beta-catenin/E-cadherin complex formation during the mechanotransduction of Saos-2 osteoblastic cells. Mol. Med. Rep..

[285] Cattaruzza M., Lattrich C., Hecker M. (2004). Focal adhesion protein zyxin is a mechanosensitive modulator of gene expression in vascular smooth muscle cells. Hypertension.

[286] Suresh Babu S., Wojtowicz A., Freichel M., Birnbaumer L., Hecker M., Cattaruzza M. (2012). Mechanism of stretch-induced activation of the mechanotransducer zyxin in vascular cells. Sci. Signal..

[287] Wang Y.X., Wang D.Y., Guo Y.C., Guo J. (2019). Zyxin: A mechanotransductor to regulate gene expression. Eur. Rev. Med. Pharmacol. Sci..

[288] Miralles F., Posern G., Zaromytidou A.I., Treisman R. (2003). Actin dynamics control SRF activity by regulation of its coactivator MAL. Cell.

[289] Zhao X.H., Laschinger C., Arora P., Szaszi K., Kapus A., McCulloch C.A. (2007). Force activates smooth muscle alpha-actin promoter activity through the Rho signaling pathway. J. Cell Sci..

[290] Vartiainen M.K., Guettler S., Larijani B., Treisman R. (2007). Nuclear actin regulates dynamic subcellular localization and activity of the SRF cofactor MAL. Science.

[291] Ho C.Y., Jaalouk D.E., Vartiainen M.K., Lammerding J. (2013). Lamin A/C and emerin regulate MKL1-SRF activity by modulating actin dynamics. Nature.

[292] Chandorkar Y., Castro Nava A., Schweizerhof S., Van Dongen M., Haraszti T., Kohler J., Zhang H., Windoffer R., Mourran A., Moller M. (2019). Cellular responses to beating hydrogels to investigate mechanotransduction. Nat. Commun..

[293] Senyo S.E., Koshman Y.E., Russell B. (2007). Stimulus interval, rate and direction differentially regulate phosphorylation for mechanotransduction in neonatal cardiac myocytes. FEBS Lett..

[294] Zhao B., Wei X., Li W., Udan R.S., Yang Q., Kim J., Xie J., Ikenoue T., Yu J., Li L. (2007). Inactivation of YAP oncoprotein by the Hippo pathway is involved in cell contact inhibition and tissue growth control. Genes Dev..

[295] Cheng M., Wu J., Li Y., Nie Y., Chen H. (2008). Activation of MAPK participates in low shear stress-induced IL-8 gene expression in endothelial cells. Clin. Biomech..

[296] Gayer C.P., Craig D.H., Flanigan T.L., Reed T.D., Cress D.E., Basson M.D. (2010). ERK regulates strain-induced migration and proliferation from different subcellular locations. J. Cell. Biochem..

[297] Gortazar A.R., Martin-Millan M., Bravo B., Plotkin L.I., Bellido T. (2013). Crosstalk between caveolin-1/extracellular signal-regulated kinase (ERK) and beta-catenin survival pathways in osteocyte mechanotransduction. J. Biol. Chem..

[298] Qin X., Li J., Sun J., Liu L., Chen D., Liu Y. (2019). Low shear stress induces ERK nuclear localization and YAP activation to control the proliferation of breast cancer cells. Biochem. Biophys. Res. Commun..

[299] Masumura T., Yamamoto K., Shimizu N., Obi S., Ando J. (2009). Shear stress increases expression of the arterial endothelial marker ephrinB2 in murine ES cells via the VEGF-Notch signaling pathways. Arterioscler. Thromb. Vasc. Biol..

[300] Fang J.S., Coon B.G., Gillis N., Chen Z., Qiu J., Chittenden T.W., Burt J.M., Schwartz M.A., Hirschi K.K. (2017). Shear-induced Notch-Cx37-p27 axis arrests endothelial cell cycle to enable arterial specification. Nat. Commun..

[301] Mack J.J., Mosqueiro T.S., Archer B.J., Jones W.M., Sunshine H., Faas G.C., Briot A., Aragon R.L., Su T., Romay M.C. (2017). NOTCH1 is a mechanosensor in adult arteries. Nat. Commun..

[302] Steinbuck M.P., Winandy S. (2018). A Review of Notch Processing With New Insights Into Ligand-Independent Notch Signaling in T-Cells. Front. Immunol..

[303] Liu Y., Xin Y., Ye F., Wang W., Lu Q., Kaplan H.J., Dean D.C. (2010). Taz-tead1 links cell-cell contact to zeb1 expression, proliferation, and dedifferentiation in retinal pigment epithelial cells. Investig. Ophthalmol. Vis. Sci..

[304] Lin K.C., Moroishi T., Meng Z., Jeong H.S., Plouffe S.W., Sekido Y., Han J., Park H.W., Guan K.L. (2017). Regulation of Hippo pathway transcription factor TEAD by p38 MAPK-induced cytoplasmic translocation. Nat. Cell Biol..

[305] Dalagiorgou G., Basdra E.K., Papavassiliou A.G. (2010). Polycystin-1: Function as a mechanosensor. Int. J. Biochem. Cell Biol..

[306] Kalogeropoulos M., Varanasi S.S., Olstad O.K., Sanderson P., Gautvik V.T., Reppe S., Francis R.M., Gautvik K.M., Birch M.A., Datta H.K. (2010). Zic1 transcription factor in bone: Neural developmental protein regulates mechanotransduction in osteocytes. FASEB J..

[307] Kook S.H., Jang Y.S., Lee J.C. (2011). Involvement of JNK-AP-1 and ERK-NF-kappaB signaling in tension-stimulated expression of type I collagen and MMP-1 in human periodontal ligament fibroblasts. J. Appl. Physiol..

[308] Nayebosadri A., Christopher L., Ji J.Y. (2012). Bayesian image analysis of dexamethasone and shear stress-induced glucocorticoid receptor intracellular movement. Ann. Biomed. Eng..

[309] Ji J.Y. (2018). Endothelial Nuclear Lamina in Mechanotransduction Under Shear Stress. Adv. Exp. Med. Biol..

[310] Yang C., Tibbitt M.W., Basta L., Anseth K.S. (2014). Mechanical memory and dosing influence stem cell fate. Nat. Mater..

[311] Sun Y., Yong K.M., Villa-Diaz L.G., Zhang X., Chen W., Philson R., Weng S., Xu H., Krebsbach P.H., Fu J. (2014). Hippo/YAP-mediated rigidity-dependent motor neuron differentiation of human pluripotent stem cells. Nat. Mater..

[312] Wei S.C., Fattet L., Tsai J.H., Guo Y., Pai V.H., Majeski H.E., Chen A.C., Sah R.L., Taylor S.S., Engler A.J. (2015). Matrix stiffness drives epithelial-mesenchymal transition and tumour metastasis through a TWIST1-G3BP2 mechanotransduction pathway. Nat. Cell Biol..

[313] Urbanski M.M., Kingsbury L., Moussouros D., Kassim I., Mehjabeen S., Paknejad N., Melendez-Vasquez C.V. (2016). Myelinating glia differentiation is regulated by extracellular matrix elasticity. Sci. Rep..

[314] Nakazawa N., Sathe A.R., Shivashankar G.V., Sheetz M.P. (2016). Matrix mechanics controls FHL2 movement to the nucleus to activate p21 expression. Proc. Natl. Acad. Sci. USA.

[315] Chen C., Wei X., Wang S., Jiao Q., Zhang Y., Du G., Wang X., Wei F., Zhang J., Wei L. (2016). Compression regulates gene expression of chondrocytes through HDAC4 nuclear relocation via PP2A-dependent HDAC4 dephosphorylation. Biochim. Biophys. Acta.

[316] Saez F., Hong N.J., Garvin J.L. (2016). Luminal flow induces NADPH oxidase 4 translocation to the nuclei of thick ascending limbs. Physiol. Rep..

[317] Enyedi B., Jelcic M., Niethammer P. (2016). The Cell Nucleus Serves as a Mechanotransducer of Tissue Damage-Induced Inflammation. Cell.

[318] Furukawa K.T., Yamashita K., Sakurai N., Ohno S. (2017). The Epithelial Circumferential Actin Belt Regulates YAP/TAZ through Nucleocytoplasmic Shuttling of Merlin. Cell Rep..

[319] Pelaez D., Acosta Torres Z., Ng T.K., Choy K.W., Pang C.P., Cheung H.S. (2017). Cardiomyogenesis of periodontal ligament-derived stem cells by dynamic tensile strain. Cell Tissue Res..

[320] Dalagiorgou G., Piperi C., Adamopoulos C., Georgopoulou U., Gargalionis A.N., Spyropoulou A., Zoi I., Nokhbehsaim M., Damanaki A., Deschner J. (2017). Mechanosensor polycystin-1 potentiates differentiation of human osteoblastic cells by upregulating Runx2 expression via induction of JAK2/STAT3 signaling axis. Cell. Mol. Life Sci..

[321] Dou C., Liu Z., Tu K., Zhang H., Chen C., Yaqoob U., Wang Y., Wen J., van Deursen J., Sicard D. (2018). P300 Acetyltransferase Mediates Stiffness-Induced Activation of Hepatic Stellate Cells Into Tumor-Promoting Myofibroblasts. Gastroenterology.

[322] Torrino S., Shen W.W., Blouin C.M., Mani S.K., Viaris de Lesegno C., Bost P., Grassart A., Koster D., Valades-Cruz C.A., Chambon V. (2018). EHD2 is a mechanotransducer connecting caveolae dynamics with gene transcription. J. Cell Biol..

[323] McMahon K.A., Wu Y., Gambin Y., Sierecki E., Tillu V.A., Hall T., Martel N., Okano S., Moradi S.V., Ruelcke J.E. (2019). Identification of intracellular cavin target proteins reveals cavin-PP1alpha interactions regulate apoptosis. Nat. Commun..

[324] Bailey K.A., Moreno E., Haj F.G., Simon S.I., Passerini A.G. (2019). Mechanoregulation of p38 activity enhances endoplasmic reticulum stress-mediated inflammation by arterial endothelium. FASEB J..

[325] Pereira D., Richert A., Medjkane S., Henon S., Weitzman J.B. (2020). Cell geometry and the cytoskeleton impact the nucleo-cytoplasmic localisation of the SMYD3 methyltransferase. Sci. Rep..

[326] Godbout E., Son D.O., Hume S., Boo S., Sarrazy V., Clement S., Kapus A., Wehrle-Haller B., Bruckner-Tuderman L., Has C. (2020). Kindlin-2 Mediates Mechanical Activation of Cardiac Myofibroblasts. Cells.

[327] Siddiqui M.Q., Badmalia M.D., Patel T.R. (2021). Bioinformatic Analysis of Structure and Function of LIM Domains of Human Zyxin Family Proteins. Int. J. Mol. Sci..

[328] Sporkova A., Ghosh S., Al-Hasani J., Hecker M. (2021). Lin11-Isl1-Mec3 Domain Proteins as Mechanotransducers in Endothelial and Vascular Smooth Muscle Cells. Front. Physiol..

[329] Tsai W.Y., Hsueh Y.Y., Chen P.Y., Hung K.S., Huang C.C. (2022). High-Frequency Ultrasound Elastography for Assessing Elastic Properties of Skin and Scars. IEEE Trans. Ultrason. Ferroelectr. Freq. Control.

[330] Park S., Chien A.L., Brown I.D., Chen J. (2023). Characterizing viscoelastic properties of human melanoma tissue using Prony series. Front. Bioeng. Biotechnol..

[331] Reiss-Zimmermann M., Streitberger K.J., Sack I., Braun J., Arlt F., Fritzsch D., Hoffmann K.T. (2015). High Resolution Imaging of Viscoelastic Properties of Intracranial Tumours by Multi-Frequency Magnetic Resonance Elastography. Clin. Neuroradiol..

[332] Dittmann F., Hirsch S., Tzschatzsch H., Guo J., Braun J., Sack I. (2016). In vivo wideband multifrequency MR elastography of the human brain and liver. Magn. Reson. Med..

[333] Antonovaite N., Hulshof L.A., Hol E.M., Wadman W.J., Iannuzzi D. (2021). Viscoelastic mapping of mouse brain tissue: Relation to structure and age. J. Mech. Behav. Biomed. Mater..

[334] Yeh W.C., Li P.C., Jeng Y.M., Hsu H.C., Kuo P.L., Li M.L., Yang P.M., Lee P.H. (2002). Elastic modulus measurements of human liver and correlation with pathology. Ultrasound Med. Biol..

[335] Petzold G., Hofer J., Ellenrieder V., Neesse A., Kunsch S. (2019). Liver Stiffness Measured by 2-Dimensional Shear Wave Elastography: Prospective Evaluation of Healthy Volunteers and Patients With Liver Cirrhosis. J. Ultrasound Med..

[336] Samani A., Zubovits J., Plewes D. (2007). Elastic moduli of normal and pathological human breast tissues: An inversion-technique-based investigation of 169 samples. Phys. Med. Biol..

[337] Zhang M., Nigwekar P., Castaneda B., Hoyt K., Joseph J.V., di Sant’Agnese A., Messing E.M., Strang J.G., Rubens D.J., Parker K.J. (2008). Quantitative characterization of viscoelastic properties of human prostate correlated with histology. Ultrasound Med. Biol..

[338] Good D.W., Stewart G.D., Hammer S., Scanlan P., Shu W., Phipps S., Reuben R., McNeill A.S. (2014). Elasticity as a biomarker for prostate cancer: A systematic review. BJU Int..

[339] Zemla J., Bobrowska J., Kubiak A., Zielinski T., Pabijan J., Pogoda K., Bobrowski P., Lekka M. (2020). Indenting soft samples (hydrogels and cells) with cantilevers possessing various shapes of probing tip. Eur. Biophys. J..

[340] Yen M.H., Chen Y.H., Liu Y.S., Lee O.K. (2020). Alteration of Young’s modulus in mesenchymal stromal cells during osteogenesis measured by atomic force microscopy. Biochem. Biophys. Res. Commun..

[341] Kasza K.E., Nakamura F., Hu S., Kollmannsberger P., Bonakdar N., Fabry B., Stossel T.P., Wang N., Weitz D.A. (2009). Filamin A is essential for active cell stiffening but not passive stiffening under external force. Biophys. J..

[342] Lekka M., Laidler P., Gil D., Lekki J., Stachura Z., Hrynkiewicz A.Z. (1999). Elasticity of normal and cancerous human bladder cells studied by scanning force microscopy. Eur. Biophys. J..

[343] Dey K., Roca E., Ramorino G., Sartore L. (2020). Progress in the mechanical modulation of cell functions in tissue engineering. Biomater. Sci..

[344] Navindaran K., Kang J.S., Moon K. (2023). Techniques for characterizing mechanical properties of soft tissues. J. Mech. Behav. Biomed. Mater..

