# Supplementary material for: The Role of Mechanotransduction in Contact Inhibition of Locomotion and Proliferation
Source: Int J Mol Sci. 2024 Feb 10;25(4):2135. doi: 10.3390/ijms25042135 (PMC10889191; doi:10.3390/ijms25042135)
Supplement: Supplementary file 1 [file ijms-25-02135-s001.zip › Table S1.pdf]

**Table S1. Cell density- and mechano-sensitive nucleocytoplasmic shuttling molecules.**

| Protein Name<br>*Gene Name* | MW<br>Human | Function | Year* | Ref. |
|---|---|---|---|---|
| Sterol regulatory element-binding protein 1<br>*SREBP1* | 1147 | Transcription factor that regulates expression of genes involved in lipid homeostasis and cholesterol biosynthesis. SREBP1 localizes in the nuclei in cells under both laminar and disturbed flows for 1 hour. After 12 hr, unlike disturbed flow, laminar flow leads to translocation of SREBP1 to the cytoplasm. | 2002 | [1] |
| Nuclear factor NF-kappa-B p105 subunit<br>*NFKB1* | 968 | Transcription factor involved in many biological processes. Cyclic tensile strain of low magnitude inhibits the IL-1b-induced nuclear translocation of the p65/p50 dimers of NF-kappa-B. | 2003 | [2-4] |
| Catenin beta-1<br>*CTNNB1* | 781 | Component of an E-cadherin/catenin adhesion complex. Also downstream component of the canonical Wnt signaling pathway. In the presence of Wnt ligand, CTNNB1 accumulates in the nucleus and acts as a coactivator for transcription factors of the TCF/LEF family, leading to activate Wnt responsive genes. Fluid shear stress in osteoblasts induces translocation of beta-catenin to the nucleus. | 2004 | [5-8] |
| Zyxin<br>*ZYX* | 572 | Adaptor protein at focal adhesion regulating cytoskeletal dynamics and signal transduction. Cyclic stretch, but not to osmotic induces dissociation of zyxin from focal adhesions and translocation to the nucleus. | 2004 | [9-11] |
| Myocardin-related transcription factor A *MRTFA* (*MAL*/*MKL1*) | 931 | Transcription coactivator that associates with the serum response factor (SRF) transcription factor to control expression of genes involved in development, morphogenesis and cell migration.<br>MRTFA binds G-actin that regulates activity of the MRTFA-SRF complex. Activity is also regulated by F-actin in the nucleus. Force application induces nuclear translocation of MRTF-A but not MRTF-B. | 2007 | [12-16] |
| Focal adhesion kinase 1<br>*PTK2* (*FAK*) | 1052 | Non-receptor protein-tyrosine kinase that regulates formation and disassembly of focal adhesion, cell migration, proliferation and apoptosis. After 30 min of 10% static strain FAK translocates to spots within and around the nuclei. | 2007 | [17] |
| Transcriptional coactivator YAP1<br>*YAP1* | 504 | YAP (Yes-associated protein) localization and activity are regulated by cell density via the Hippo pathway. | 2007 | [18] |
| Phosphorylated mitogen-activated protein kinase 3/1 (ERK1/2), mitogen-activated protein kinase 8/9 (JNK1/2), mitogen-activated protein kinase 11~14 (p38 MAPK)<br>*MAPK3/1*,<br>*MAPK8/9*,<br>*MAPK14* | 379/360<br>427/424<br>360~367 | Serine/threonine kinases in the MAP kinase signal transduction pathway. Low shear stress to endothelial cells induces phosphorylation and translocation of ERK1/2, p38 MAPK, and JNK1/2 from the cytoplasm into the nucleus. | 2008 | [19-22] |
| Notch intracellular domain | 2555 (intracell | Receptor for membrane-bound ligands Jagged-1, Jagged-2, and Delta-1 to regulate cell-fate determination. Shear stress | 2009 | [23-26] |

| | | | | |
|---|---|---|---|---|
| | ular domain : 802) | induces cleavage of Notch and translocation of the Notch intracellular domain into the nucleus. | | |
| Transcriptional enhancer factor TEF-1/WW domain-containing transcription regulator protein 1<br>*TEAD1*/<br>*WWTR1*(*TAZ*) | 426<br>400 | TEAD1 is transcription factor which plays a key role in the Hippo signaling pathway. WWTR1 (TAZ) is a transcriptional coactivator that binds to TEAD1. Loss of cell-cell contact leads to nuclear translocation of TAZ and TEAD1 | 2010 | [27, 28] |
| Polycystin- 1<br>*PKD1* | 4303 (~200 amino acid C-terminal cytoplasmic tail) | Form complex with PKD2 to act as a heteromeric calcium-permeable ion channel that is activated by a Wnt family member. Upon mechanical stimulation, the C-terminal cytoplasmic tail (CT) of PKD1 is proteolytically cleaved and released from the membrane, being translocated to the nucleus. Nuclear translocated CT interacts with several transcription factors such as AP-1 and STAT3. | 2010 | [29] |
| Zinc finger protein ZIC 1<br>*ZIC1* | 447 | Transcriptional activator. Fluid shear stress induces nuclear localization of Zic1 in osteocytes. | 2010 | [30] |
| Transcription factor Jun (Activator protein-1/ AP-1), transcription factor p65 (RelA/p65), and nuclear factor NF-kappa-B p105 subunit (cleaved into p50 subunit)<br>*JUN*<br>*NFKB1*<br>*RALA*<br>*NFKB1* | 331<br>551<br>968 (p50: 433) | Transcription factor that heterodimerizes with proteins of the FOS family to form an AP-1 transcription complex to induce transcription. Tensile force increases the nuclear translocation of c-Jun, RalA, and p50. | 2011 | [31] |
| Transcriptional coactivator YAP1/WW domain-containing transcription regulator protein 1<br>*YAP1*/<br>*WWTR1*(*TAZ*) | 504/400 | Transcriptional coactivator and corepressor in the Hippo signaling pathway. YAP/TAZ translocate between the nucleus and cytoplasm depending on substrate rigidity and actomyosin contraction. The translocation is also cell density-dependent. | 2011 | [32] |
| Glucocorticoid receptor<br>*NR3C1* | 777 | Transcription factor that binds to glucocorticoid response elements of nuclear and mitochondrial DNA. Shear stress induces nuclear translocation of glucocorticoid receptor. | 2012 | [33, 34] |
| Retinoic acid receptor gamma<br>*RARG* | 454 | Stiff substrates drive translocation of the transcription factor retinoic acid receptor gamma into the nucleus to regulate lamin A/C transcription. | 2013 | [35] |
| Runt-related transcription factor 2<br>*RUNX2* | 521 | Transcription factor involved in osteoblastic differentiation and skeletal morphogenesis. YAP and RUNX2 are located in the nuclei of hMSCs cultured on tissue culture polystyrene plates. YAP and RUNX2 are located in the cytoplasm of hMSCs cultured strictly on soft hydrogels. | 2014 | [36] |

| | | | | |
|---|---|---|---|---|
| Mothers against decapentaplegic homolog 1/5/8, 2/3<br>*SMAD1/5/8, 2/3* | 465/465/467<br>467/425 | Transcriptional modulator. Substrate rigidity regulates nucleocytoplasmic shuttling of SMADs. | 2014 | [37] |
| Twist-related protein 1<br>TWIST1 | 202 | Acts as a transcriptional regulator. Inhibits myogenesis by sequestrating E proteins, inhibiting Transcriptional regulator. Matrix stiffness promotes nuclear translocation of TWIST1 by releasing TWIST1 from G3BP2 in the cytoplasm. | 2015 | [38] |
| Oligodendrocyte transcription factor 1, Oligo1, Class B basic helix-loop-helix protein 6 (bHLHb6), Class E basic helix-loop-helix protein 21 (bHLHe21)<br>OLIG1, BHLHB6, BHLHE21 | 271 | Oligodendrocyte transcription factor 1 (Olig1) is a key regulator of oligodendrocyte development and translocated to the nucleus on stiff but not on soft substrates. During oligodendrocyte differentiation, Olig1 is phosphorylated and translocated from the nucleus to the cytoplasm. Olig1 cytoplasmic localization depends on its phosphorylation on serine 138 by PKA. | 2016 | [39] |
| Four and a half LIM domains protein 2<br>FHL2 | 279 | Transcriptional coactivator and a scaffold protein in cell adhesions. Matrix mechanics regulates nucleocytoplasmic shuttling of FHL2. Unlike YAP1, soft substrates stimulate FHL2 transport to the nucleus. | 2016 | [40] |
| Histone deacetylase 4<br>HDAC4 | 1084 | Deacetylate lysine residues on the core histones (H2A, H2B, H3 and H4) to regulate epigenetic changes in transcriptional regulation.<br>Mechanical compression induces HDAC4 nuclear import. | 2016 | [41] |
| NADPH oxidase 4<br>NOX4 | 578 | Produce superoxide in the nucleus and regulate gene expression. Luminal flow induces Nox4 translocation to the nucleus. | 2016 | [42] |
| Cytosolic phospholipase A2 (cPLA2)<br>PLA2G4A | 749 | Calcium-dependent phospholipase and lysophospholipase. Osmotic swelling of cells and their nuclei activates cPla2 by translocating it from the nucleoplasm to the nuclear envelope. | 2016 | [43] |
| Merlin<br>*NF2* | 595 | Tumor suppressor protein that interacts with E-cadherin and F-actin. At low cell density, merlin is associated with E-cadherin. At high density, contraction of the circumferential actin belt releases merlin from E-cadherin and then merlin is imported into the nucleus to export nuclear YAP/TAZ into the cytoplasm. | 2017 | [44] |
| Cardiac-specific transcription factors GATA4, MEF2C, and NKX2-5<br>*GATA4*<br>*MEF2C*<br>*NKX2-5* | 442<br>473<br>324 | Tensile strain induces translocation of cardiac-specific transcription factors GATA4, MEF2C and Nkx2.5, and induced expression of the sarcomeric actin and cardiac troponin T proteins. | 2017 | [45] |
| Phosphorylated signal transducer and activator of transcription 3<br>*STAT3* | 770 | Signal transducer and transcription activator. Mechanical stretching induces interaction of cleaved C-terminal tail of PKD1 with phosphorylated JAK2 in human osteoblastic cells. The active JAK2 phosphorylates STAT3 (active) that translocates to the nucleus to induce gene expression. | 2017 | [46] |
| Histone acetyltransferase p300 | 2414 | Histone acetyltransferase that regulates transcription by remodeling chromatin. Substrate stiffness regulates AKT | 2018 | [47] |

| | | | | |
|---|---|---|---|---|
| *EP300* | | signaling via RhoA to induce phosphorylation of p300 that translocates to the nucleus in Hepatic stellate cells. | | |
| EH domain-containing protein 2 *EHD2* | 543 | ATPase that binds membrane. cyclic stretching and hypo-osmotic shock induce release of EHD2 from caveolae, SUMOylation, and translocation to the nucleus to regulate gene expression. | 2018 | [48] |
| Caveolae-associated protein 1 *CAVIN1* | 390 | Core component of the CAVIN in the caveolae. Also called polymerase I and transcript release factor that promotes ribosomal RNA transcription. Mild hypo-osmotic stress induces translocation of a large portion of the plasma membrane GFP-cavin-1 to the cytosol and the nucleus. | 2019 | [49] |
| ANKHD1 and ANKRD17 | | Mask family proteins contain two ankyrin repeat domains that bind Yki/YAP as well as a conserved nuclear localisation sequence (NLS) and nuclear export sequence (NES), suggesting a role in nucleo-cytoplasmic transport. | 2019 | [50] |
| X-box-binding protein 1, interferon regulatory factor 1 *XBP1*, *IRF1* | 261 325 | Transcription factors. Low shear stress induces temporal rise in p38 phosphorylation that activates the nuclear translocation of XBP1 and IFN regulatory factor 1. | 2019 | [51] |
| Histone-lysine N-methyltransferase SMYD3 *SMYD3* | 428 | Methylates Lys-4 of histone H3, inducing di- and tri-methylation, but not monomethylation. Also methylates Lys-5 of histone H4. Unlike YAP/TAZ, disruption of actomyosin contraction promote nuclear localization of SMYD3. | 2020 | [52] |
| Fermitin family homolog 2 (Kindlin-2) *FERMT2* | 680 | Scaffolding protein that enhances integrin activation mediated by talins. Stressing fibroblasts using ferromagnetic microbeads, stretchable silicone membranes, and cell contraction induced by PAR-1 agonist promote nuclear translocation of kindlin-2. | 2020 | [53] |
| Lipoma preferred partner LPP | 612 | The nearest relative of zyxin among the Lin11-Isl1-Mec3 (LIM) domain-containing proteins. Similar to zyxin, LPP is localized to focal adhesions and cell–cell junctions. Upon exposure of cells to biomechanical deformation, LPP only transiently translocates to the nucleus to function as a transcriptional co-activator. | 2021 | [54, 55] |
| Importin 7 *IPO7* | 1038 | Ran-dependent nuclear transport receptor. Form YAP/Imp7 complex to respond to mechanical cues. YAP is as a dominant cargo of Imp7, restricting the Imp7 binding to other Imp7 cargoes such as Smad3 and Erk2. | 2022 | [56] |
| Ubiquitin-conjugating enzyme E2 A/B *UBE2A/B (RAD6A/B)* | 152 | Ubiquitin-conjugating enzyme E2 that regulates transcription by catalyzing the monoubiquitination of histone H2B at 'Lys-120' to form H2BK120ub1. Cell density- and force-dependent nucleocytoplasmic translocation. | 2023 | [57] |
| Mothers against decapentaplegic homolog 4 SMAD4 | 552 | Component of the heterotrimeric SMAD2/SMAD3/SMAD4 complex that forms in the nucleus to stimulate transcription. Translocate from the nucleus to the cytosol at high-density. | 2023 | [58] |
| Prostaglandin E synthase 3 *PTGES3* | 160 | Molecular chaperone that localizes to genomic response elements in a hormone-dependent manner and disrupts receptor-mediated transcriptional activation. Translocate from the nucleus to the cytosol at high-density. | 2023 | [58] |
| Protein mono-ADP-ribosyltransferase TIPARP *TIPARP* | 657 | ADP-ribosyltransferase that mediates mono-ADP-ribosylation of aspartate, cysteine, and glutamate residues on target proteins. Negative regulator of aryl hydrocarbon receptor (AHR), a ligand-activated transcription factor, by mediating | 2023 | [58] |

| | | | | |
|---|---|---|---|---|
| | | mono-ADP-ribosylation of AHR, leading to inhibit AHR activity. Translocate from the nucleus to the cytosol at high-density. | | |
| Core-binding factor subunit beta *CBFB* | 182 | Forms the heterodimeric complex core-binding factor (CBF) with RUNX family proteins (RUNX1, RUNX2, and RUNX3). RUNX members modulate the transcription of their target genes. Translocate from the nucleus to the cytosol at high-density. | 2023 | [58] |

* In chronological order.

**References**


1. Liu, Y.; Chen, B. P.; Lu, M.; Zhu, Y.; Stemerman, M. B.; Chien, S.; Shyy, J. Y., Shear stress activation of SREBP1 in endothelial cells is mediated by integrins. *Arterioscler Thromb Vasc Biol* **2002,** 22, (1), 76-81.
2. Deschner, J.; Hofman, C. R.; Piesco, N. P.; Agarwal, S., Signal transduction by mechanical strain in chondrocytes. *Curr Opin Clin Nutr Metab Care* **2003,** 6, (3), 289-93.
3. Chen, N. X.; Geist, D. J.; Genetos, D. C.; Pavalko, F. M.; Duncan, R. L., Fluid shear-induced NFkappaB translocation in osteoblasts is mediated by intracellular calcium release. *Bone* **2003,** 33, (3), 399-410.
4. Young, S. R.; Gerard-O'Riley, R.; Harrington, M.; Pavalko, F. M., Activation of NF-kappaB by fluid shear stress, but not TNF-alpha, requires focal adhesion kinase in osteoblasts. *Bone* **2010,** 47, (1), 74-82.
5. Norvell, S. M.; Alvarez, M.; Bidwell, J. P.; Pavalko, F. M., Fluid shear stress induces beta-catenin signaling in osteoblasts. *Calcif Tissue Int* **2004,** 75, (5), 396-404.
6. Yang, Z.; Bidwell, J. P.; Young, S. R.; Gerard-O'Riley, R.; Wang, H.; Pavalko, F. M., Nmp4/CIZ inhibits mechanically induced beta-catenin signaling activity in osteoblasts. *J Cell Physiol* **2010,** 223, (2), 435-41.
7. Liu, S.; Zhou, F.; Shen, Y.; Zhang, Y.; Yin, H.; Zeng, Y.; Liu, J.; Yan, Z.; Liu, X., Fluid shear stress induces epithelial-mesenchymal transition (EMT) in Hep-2 cells. *Oncotarget* **2016,** 7, (22), 32876-92.
8. Li, F. F.; Zhang, B.; Cui, J. H.; Chen, F. L.; Ding, Y.; Feng, X., Alterations in beta-catenin/E-cadherin complex formation during the mechanotransduction of Saos-2 osteoblastic cells. *Mol Med Rep* **2018,** 18, (2), 1495-1503.
9. Cattaruzza, M.; Lattrich, C.; Hecker, M., Focal adhesion protein zyxin is a mechanosensitive modulator of gene expression in vascular smooth muscle cells. *Hypertension* **2004,** 43, (4), 726-30.
10. Suresh Babu, S.; Wojtowicz, A.; Freichel, M.; Birnbaumer, L.; Hecker, M.; Cattaruzza, M., Mechanism of stretch-induced activation of the mechanotransducer zyxin in vascular cells. *Sci Signal* **2012,** 5, (254), ra91.
11. Wang, Y. X.; Wang, D. Y.; Guo, Y. C.; Guo, J., Zyxin: a mechanotransductor to regulate gene expression. *Eur Rev Med Pharmacol Sci* **2019,** 23, (1), 413-425.
12. Miralles, F.; Posern, G.; Zaromytidou, A. I.; Treisman, R., Actin dynamics control SRF activity by regulation of its coactivator MAL. *Cell* **2003,** 113, (3), 329-42.
13. Zhao, X. H.; Laschinger, C.; Arora, P.; Szaszi, K.; Kapus, A.; McCulloch, C. A., Force activates smooth muscle alpha-actin promoter activity through the Rho signaling pathway. *J Cell Sci* **2007,** 120, (Pt 10), 1801-9.

14. Vartiainen, M. K.; Guettler, S.; Larijani, B.; Treisman, R., Nuclear actin regulates dynamic subcellular localization and activity of the SRF cofactor MAL. *Science* **2007,** 316, (5832), 1749-52.
15. Ho, C. Y.; Jaalouk, D. E.; Vartiainen, M. K.; Lammerding, J., Lamin A/C and emerin regulate MKL1-SRF activity by modulating actin dynamics. *Nature* **2013,** 497, (7450), 507-11.
16. Chandorkar, Y.; Castro Nava, A.; Schweizerhof, S.; Van Dongen, M.; Haraszti, T.; Kohler, J.; Zhang, H.; Windoffer, R.; Mourran, A.; Moller, M.; De Laporte, L., Cellular responses to beating hydrogels to investigate mechanotransduction. *Nat Commun* **2019,** 10, (1), 4027.
17. Senyo, S. E.; Koshman, Y. E.; Russell, B., Stimulus interval, rate and direction differentially regulate phosphorylation for mechanotransduction in neonatal cardiac myocytes. *FEBS Lett* **2007,** 581, (22), 4241-7.
18. Zhao, B.; Wei, X.; Li, W.; Udan, R. S.; Yang, Q.; Kim, J.; Xie, J.; Ikenoue, T.; Yu, J.; Li, L.; Zheng, P.; Ye, K.; Chinnaiyan, A.; Halder, G.; Lai, Z. C.; Guan, K. L., Inactivation of YAP oncoprotein by the Hippo pathway is involved in cell contact inhibition and tissue growth control. *Genes Dev* **2007,** 21, (21), 2747-61.
19. Cheng, M.; Wu, J.; Li, Y.; Nie, Y.; Chen, H., Activation of MAPK participates in low shear stress-induced IL-8 gene expression in endothelial cells. *Clin Biomech (Bristol, Avon)* **2008,** 23 Suppl 1, S96-S103.
20. Gayer, C. P.; Craig, D. H.; Flanigan, T. L.; Reed, T. D.; Cress, D. E.; Basson, M. D., ERK regulates strain-induced migration and proliferation from different subcellular locations. *J Cell Biochem* **2010,** 109, (4), 711-25.
21. Gortazar, A. R.; Martin-Millan, M.; Bravo, B.; Plotkin, L. I.; Bellido, T., Crosstalk between caveolin-1/extracellular signal-regulated kinase (ERK) and beta-catenin survival pathways in osteocyte mechanotransduction. *J Biol Chem* **2013,** 288, (12), 8168-75.
22. Qin, X.; Li, J.; Sun, J.; Liu, L.; Chen, D.; Liu, Y., Low shear stress induces ERK nuclear localization and YAP activation to control the proliferation of breast cancer cells. *Biochem Biophys Res Commun* **2019,** 510, (2), 219-223.
23. Masumura, T.; Yamamoto, K.; Shimizu, N.; Obi, S.; Ando, J., Shear stress increases expression of the arterial endothelial marker ephrinB2 in murine ES cells via the VEGF-Notch signaling pathways. *Arterioscler Thromb Vasc Biol* **2009,** 29, (12), 2125-31.
24. Fang, J. S.; Coon, B. G.; Gillis, N.; Chen, Z.; Qiu, J.; Chittenden, T. W.; Burt, J. M.; Schwartz, M. A.; Hirschi, K. K., Shear-induced Notch-Cx37-p27 axis arrests endothelial cell cycle to enable arterial specification. *Nat Commun* **2017,** 8, (1), 2149.
25. Mack, J. J.; Mosqueiro, T. S.; Archer, B. J.; Jones, W. M.; Sunshine, H.; Faas, G. C.; Briot, A.; Aragon, R. L.; Su, T.; Romay, M. C.; McDonald, A. I.; Kuo, C. H.; Lizama, C. O.; Lane, T. F.; Zovein, A. C.; Fang, Y.; Tarling, E. J.; de Aguiar Vallim, T. Q.; Navab, M.; Fogelman, A. M.; Bouchard, L. S.; Iruela-Arispe, M. L., NOTCH1 is a mechanosensor in adult arteries. *Nat Commun* **2017,** 8, (1), 1620.
26. Steinbuck, M. P.; Winandy, S., A Review of Notch Processing With New Insights Into Ligand-Independent Notch Signaling in T-Cells. *Front Immunol* **2018,** 9, 1230.
27. Liu, Y.; Xin, Y.; Ye, F.; Wang, W.; Lu, Q.; Kaplan, H. J.; Dean, D. C., Taz-tead1 links cell-cell contact to zeb1 expression, proliferation, and dedifferentiation in retinal pigment epithelial cells. *Invest Ophthalmol Vis Sci* **2010,** 51, (7), 3372-8.

28. Lin, K. C.; Moroishi, T.; Meng, Z.; Jeong, H. S.; Plouffe, S. W.; Sekido, Y.; Han, J.; Park, H. W.; Guan, K. L., Regulation of Hippo pathway transcription factor TEAD by p38 MAPK-induced cytoplasmic translocation. *Nat Cell Biol* **2017,** 19, (8), 996-1002.
29. Dalagiorgou, G.; Basdra, E. K.; Papavassiliou, A. G., Polycystin-1: function as a mechanosensor. *Int J Biochem Cell Biol* **2010,** 42, (10), 1610-3.
30. Kalogeropoulos, M.; Varanasi, S. S.; Olstad, O. K.; Sanderson, P.; Gautvik, V. T.; Reppe, S.; Francis, R. M.; Gautvik, K. M.; Birch, M. A.; Datta, H. K., Zic1 transcription factor in bone: neural developmental protein regulates mechanotransduction in osteocytes. *FASEB J* **2010,** 24, (8), 2893-903.
31. Kook, S. H.; Jang, Y. S.; Lee, J. C., Involvement of JNK-AP-1 and ERK-NF-kappaB signaling in tension-stimulated expression of type I collagen and MMP-1 in human periodontal ligament fibroblasts. *J Appl Physiol (1985)* **2011,** 111, (6), 1575-83.
32. Dupont, S.; Morsut, L.; Aragona, M.; Enzo, E.; Giulitti, S.; Cordenonsi, M.; Zanconato, F.; Le Digabel, J.; Forcato, M.; Bicciato, S.; Elvassore, N.; Piccolo, S., Role of YAP/TAZ in mechanotransduction. *Nature* **2011,** 474, (7350), 179-83.
33. Nayebosadri, A.; Christopher, L.; Ji, J. Y., Bayesian image analysis of dexamethasone and shear stress-induced glucocorticoid receptor intracellular movement. *Ann Biomed Eng* **2012,** 40, (7), 1508-19.
34. Ji, J. Y., Endothelial Nuclear Lamina in Mechanotransduction Under Shear Stress. *Adv Exp Med Biol* **2018,** 1097, 83-104.
35. Swift, J.; Ivanovska, I. L.; Buxboim, A.; Harada, T.; Dingal, P. C.; Pinter, J.; Pajerowski, J. D.; Spinler, K. R.; Shin, J. W.; Tewari, M.; Rehfeldt, F.; Speicher, D. W.; Discher, D. E., Nuclear lamin-A scales with tissue stiffness and enhances matrix-directed differentiation. *Science* **2013,** 341, (6149), 1240104.
36. Yang, C.; Tibbitt, M. W.; Basta, L.; Anseth, K. S., Mechanical memory and dosing influence stem cell fate. *Nat Mater* **2014,** 13, (6), 645-52.
37. Sun, Y.; Yong, K. M.; Villa-Diaz, L. G.; Zhang, X.; Chen, W.; Philson, R.; Weng, S.; Xu, H.; Krebsbach, P. H.; Fu, J., Hippo/YAP-mediated rigidity-dependent motor neuron differentiation of human pluripotent stem cells. *Nat Mater* **2014,** 13, (6), 599-604.
38. Wei, S. C.; Fattet, L.; Tsai, J. H.; Guo, Y.; Pai, V. H.; Majeski, H. E.; Chen, A. C.; Sah, R. L.; Taylor, S. S.; Engler, A. J.; Yang, J., Matrix stiffness drives epithelial-mesenchymal transition and tumour metastasis through a TWIST1-G3BP2 mechanotransduction pathway. *Nat Cell Biol* **2015,** 17, (5), 678-88.
39. Urbanski, M. M.; Kingsbury, L.; Moussouros, D.; Kassim, I.; Mehjabeen, S.; Paknejad, N.; Melendez-Vasquez, C. V., Myelinating glia differentiation is regulated by extracellular matrix elasticity. *Sci Rep* **2016,** 6, 33751.
40. Nakazawa, N.; Sathe, A. R.; Shivashankar, G. V.; Sheetz, M. P., Matrix mechanics controls FHL2 movement to the nucleus to activate p21 expression. *Proc Natl Acad Sci U S A* **2016,** 113, (44), E6813-E6822.
41. Chen, C.; Wei, X.; Wang, S.; Jiao, Q.; Zhang, Y.; Du, G.; Wang, X.; Wei, F.; Zhang, J.; Wei, L., Compression regulates gene expression of chondrocytes through HDAC4 nuclear relocation via PP2A-dependent HDAC4 dephosphorylation. *Biochim Biophys Acta* **2016,** 1863, (7 Pt A), 1633-42.
42. Saez, F.; Hong, N. J.; Garvin, J. L., Luminal flow induces NADPH oxidase 4 translocation to the nuclei of thick ascending limbs. *Physiol Rep* **2016,** 4, (6).

43. Enyedi, B.; Jelcic, M.; Niethammer, P., The Cell Nucleus Serves as a Mechanotransducer of Tissue Damage-Induced Inflammation. *Cell* **2016,** 165, (5), 1160-70.
44. Furukawa, K. T.; Yamashita, K.; Sakurai, N.; Ohno, S., The Epithelial Circumferential Actin Belt Regulates YAP/TAZ through Nucleocytoplasmic Shuttling of Merlin. *Cell Rep* **2017,** 20, (6), 1435-1447.
45. Pelaez, D.; Acosta Torres, Z.; Ng, T. K.; Choy, K. W.; Pang, C. P.; Cheung, H. S., Cardiomyogenesis of periodontal ligament-derived stem cells by dynamic tensile strain. *Cell Tissue Res* **2017,** 367, (2), 229-241.
46. Dalagiorgou, G.; Piperi, C.; Adamopoulos, C.; Georgopoulou, U.; Gargalionis, A. N.; Spyropoulou, A.; Zoi, I.; Nokhbehsaim, M.; Damanaki, A.; Deschner, J.; Basdra, E. K.; Papavassiliou, A. G., Mechanosensor polycystin-1 potentiates differentiation of human osteoblastic cells by upregulating Runx2 expression via induction of JAK2/STAT3 signaling axis. *Cell Mol Life Sci* **2017,** 74, (5), 921-936.
47. Dou, C.; Liu, Z.; Tu, K.; Zhang, H.; Chen, C.; Yaqoob, U.; Wang, Y.; Wen, J.; van Deursen, J.; Sicard, D.; Tschumperlin, D.; Zou, H.; Huang, W. C.; Urrutia, R.; Shah, V. H.; Kang, N., P300 Acetyltransferase Mediates Stiffness-Induced Activation of Hepatic Stellate Cells Into Tumor-Promoting Myofibroblasts. *Gastroenterology* **2018,** 154, (8), 2209-2221 e14.
48. Torrino, S.; Shen, W. W.; Blouin, C. M.; Mani, S. K.; Viaris de Lesegno, C.; Bost, P.; Grassart, A.; Koster, D.; Valades-Cruz, C. A.; Chambon, V.; Johannes, L.; Pierobon, P.; Soumelis, V.; Coirault, C.; Vassilopoulos, S.; Lamaze, C., EHD2 is a mechanotransducer connecting caveolae dynamics with gene transcription. *J Cell Biol* **2018**.
49. McMahon, K. A.; Wu, Y.; Gambin, Y.; Sierecki, E.; Tillu, V. A.; Hall, T.; Martel, N.; Okano, S.; Moradi, S. V.; Ruelcke, J. E.; Ferguson, C.; Yap, A. S.; Alexandrov, K.; Hill, M. M.; Parton, R. G., Identification of intracellular cavin target proteins reveals cavin-PP1alpha interactions regulate apoptosis. *Nat Commun* **2019,** 10, (1), 3279.
50. Sidor, C.; Borreguero-Munoz, N.; Fletcher, G. C.; Elbediwy, A.; Guillermin, O.; Thompson, B. J., Mask family proteins ANKHD1 and ANKRD17 regulate YAP nuclear import and stability. *Elife* **2019,** 8.
51. Bailey, K. A.; Moreno, E.; Haj, F. G.; Simon, S. I.; Passerini, A. G., Mechanoregulation of p38 activity enhances endoplasmic reticulum stress-mediated inflammation by arterial endothelium. *FASEB J* **2019,** 33, (11), 12888-12899.
52. Pereira, D.; Richert, A.; Medjkane, S.; Henon, S.; Weitzman, J. B., Cell geometry and the cytoskeleton impact the nucleo-cytoplasmic localisation of the SMYD3 methyltransferase. *Sci Rep* **2020,** 10, (1), 20598.
53. Godbout, E.; Son, D. O.; Hume, S.; Boo, S.; Sarrazy, V.; Clement, S.; Kapus, A.; Wehrle-Haller, B.; Bruckner-Tuderman, L.; Has, C.; Hinz, B., Kindlin-2 Mediates Mechanical Activation of Cardiac Myofibroblasts. *Cells* **2020,** 9, (12).
54. Siddiqui, M. Q.; Badmalia, M. D.; Patel, T. R., Bioinformatic Analysis of Structure and Function of LIM Domains of Human Zyxin Family Proteins. *Int J Mol Sci* **2021,** 22, (5).
55. Sporkova, A.; Ghosh, S.; Al-Hasani, J.; Hecker, M., Lin11-Isl1-Mec3 Domain Proteins as Mechanotransducers in Endothelial and Vascular Smooth Muscle Cells. *Front Physiol* **2021,** 12, 769321.
56. Garcia-Garcia, M.; Sanchez-Perales, S.; Jarabo, P.; Calvo, E.; Huyton, T.; Fu, L.; Ng, S. C.; Sotodosos-Alonso, L.; Vazquez, J.; Casas-Tinto, S.; Gorlich, D.; Echarri, A.; Del

Pozo, M. A., Mechanical control of nuclear import by Importin-7 is regulated by its dominant cargo YAP. *Nat Commun* **2022,** 13, (1), 1174.
57. Feng, M.; Wang, J.; Li, K.; Nakamura, F., UBE2A/B is the trans-acting factor mediating mechanotransduction and contact inhibition. *Biochem J* **2023,** 480, (20), 1659-1674.
58. Li, K.; Li, Y.; Nakamura, F., Identification and partial characterization of new cell density-dependent nucleocytoplasmic shuttling proteins and open chromatin. *Sci Rep* **2023,** 13, (1), 21723.
