# Supplementary material for: The Role of Mechanotransduction in Contact Inhibition of Locomotion and Proliferation
Source: Int J Mol Sci. 2024 Feb 10;25(4):2135. doi: 10.3390/ijms25042135 (PMC10889191; doi:10.3390/ijms25042135)
Supplement: Supplementary file 1 [file ijms-25-02135-s001.zip › Table S2.pdf]

Table S2. Mechanical properties of human tissues and cells.

Human Tissue	Normal	Tumor	Methods	Ref.
Skin	healthy skin:<br>12.8 ± 5.4 kPa<br>Normal: 2~3 kPa	Scar:<br>74.8 ± 26.8 kPa<br>Melanoma tissue: 25.58 ± 5.96 kPa	High-Frequency Ultrasound Elastography, Indentation-based mechanical analyzer	[1]<br>[2]
Brain	10-25 Hz: 0.62 ± 0.08 kPa,<br>25-35 Hz: 1.56 ± 0.16 kPa,<br>40-50 Hz: 2.18 ± 0.20 kPa<br>*Brain stiffness increases with age. Also regional variations of stiffnesses.	0.87 - 1.95 kPa (30~60 Hz)	Multi-Frequency Magnetic Resonance Elastography	[3-5]
Liver	0.6-0.7 kPa<br>4.93 ± 0.83 kPa	Fibrosis: 0.7-1.8 kPa<br>(Cirrhosis: 13.29 ± 3.27 kPa)	Compression<br>Two-dimensional shear wave elastography	[6, 7]
Colorectal tissue	0.936 kPa<br>(0.374-7.33)	7.51 kPa<br>(1.08-68.0)	Tactile sensor.	[8]
Breast	Normal fibroglandular tissue<br>3.24+/-0.61 kPa	High-grade invasive ductal carcinoma<br>42.53+/-12.47 kPa	Indentation technique	[9]
Prostate	15.9 ± 5.9 kPa	40.4 ± 15.7 kPa	Mechanical device	[10, 11]

Cell	Elastic modulus	Methods	Ref.
Neuron	0.03-0.97 kPa	Atomic force microscopy	[5]
non-malignant ureter cell (HCV29), urinary bladder carcinoma cell (HT1376)	HCV29: 8.19 ± 0.46 kPa<br>HT1376: 2.47 ± 0.25 kPa	Atomic force microscopy	[12]
Human mesenchymal stem cells (MCSs)	Undifferentiated MSCs: 2.4 ± 0.64 kPa<br>Osteogenic differentiating MSCs: 12.28 ± 1.85 kPa	Atomic force microscopy	[13]
Human melanoma cell	~0.25 kPa (no FLNA)<br>~0.4 kPa (with FLNA)	Magnetic twisting cytometry	[14]

Human epithelial cells	5-18 kPa (normal)<br>0.7-2.1 kPa (cancerous)	Scanning force microscopy	[15]

Stiffness can be assessed using different methods, including rheometer, atomic force microscopy (AFM), magnetic resonance elastography, and indentation devices [16]. However, it's essential to consider that the measured stiffness can vary significantly based on the instrument used, the geometrical structure of the specimen, and experimental conditions such as the cantilever geometry for AFM, sample-instrument adhesion, and operator variability. Therefore, when comparing stiffness values from independent studies, caution should be exercised to account for these potential sources of variation [17].

References

1. Tsai, W. Y.; Hsueh, Y. Y.; Chen, P. Y.; Hung, K. S.; Huang, C. C., High-Frequency Ultrasound Elastography for Assessing Elastic Properties of Skin and Scars. *IEEE Trans Ultrason Ferroelectr Freq Control* **2022,** 69, (6), 1871-1880.
2. Park, S.; Chien, A. L.; Brown, I. D.; Chen, J., Characterizing viscoelastic properties of human melanoma tissue using Prony series. *Front Bioeng Biotechnol* **2023,** 11, 1162880.
3. Reiss-Zimmermann, M.; Streitberger, K. J.; Sack, I.; Braun, J.; Arlt, F.; Fritzsch, D.; Hoffmann, K. T., High Resolution Imaging of Viscoelastic Properties of Intracranial Tumours by Multi-Frequency Magnetic Resonance Elastography. *Clin Neuroradiol* **2015,** 25, (4), 371-8.
4. Dittmann, F.; Hirsch, S.; Tzschatzsch, H.; Guo, J.; Braun, J.; Sack, I., In vivo wideband multifrequency MR elastography of the human brain and liver. *Magn Reson Med* **2016,** 76, (4), 1116-26.
5. Antonovaite, N.; Hulshof, L. A.; Hol, E. M.; Wadman, W. J.; Iannuzzi, D., Viscoelastic mapping of mouse brain tissue: Relation to structure and age. *J Mech Behav Biomed Mater* **2021,** 113, 104159.
6. Yeh, W. C.; Li, P. C.; Jeng, Y. M.; Hsu, H. C.; Kuo, P. L.; Li, M. L.; Yang, P. M.; Lee, P. H., Elastic modulus measurements of human liver and correlation with pathology. *Ultrasound Med Biol* **2002,** 28, (4), 467-74.
7. Petzold, G.; Hofer, J.; Ellenrieder, V.; Neesse, A.; Kunsch, S., Liver Stiffness Measured by 2-Dimensional Shear Wave Elastography: Prospective Evaluation of Healthy Volunteers and Patients With Liver Cirrhosis. *J Ultrasound Med* **2019,** 38, (7), 1769-1777.
8. Kawano, S.; Kojima, M.; Higuchi, Y.; Sugimoto, M.; Ikeda, K.; Sakuyama, N.; Takahashi, S.; Hayashi, R.; Ochiai, A.; Saito, N., Assessment of elasticity of colorectal cancer tissue, clinical utility, pathological and phenotypical relevance. *Cancer Sci* **2015,** 106, (9), 1232-9.
9. Samani, A.; Zubovits, J.; Plewes, D., Elastic moduli of normal and pathological human breast tissues: an inversion-technique-based investigation of 169 samples. *Phys Med Biol* **2007,** 52, (6), 1565-76.
10. Zhang, M.; Nigwekar, P.; Castaneda, B.; Hoyt, K.; Joseph, J. V.; di Sant'Agnese, A.; Messing, E. M.; Strang, J. G.; Rubens, D. J.; Parker, K. J., Quantitative characterization of viscoelastic properties of human prostate correlated with histology. *Ultrasound Med Biol* **2008,** 34, (7), 1033-42.
11. Good, D. W.; Stewart, G. D.; Hammer, S.; Scanlan, P.; Shu, W.; Phipps, S.; Reuben, R.; McNeill, A. S., Elasticity as a biomarker for prostate cancer: a systematic review. *BJU Int* **2014,** 113, (4), 523-34.
12. Zemla, J.; Bobrowska, J.; Kubiak, A.; Zielinski, T.; Pabijan, J.; Pogoda, K.; Bobrowski, P.; Lekka, M., Indenting soft samples (hydrogels and cells) with cantilevers possessing various shapes of probing tip. *Eur Biophys J* **2020,** 49, (6), 485-495.

13. Yen, M. H.; Chen, Y. H.; Liu, Y. S.; Lee, O. K., Alteration of Young's modulus in mesenchymal stromal cells during osteogenesis measured by atomic force microscopy. *Biochem Biophys Res Commun* **2020,** 526, (3), 827-832.
14. Kasza, K. E.; Nakamura, F.; Hu, S.; Kollmannsberger, P.; Bonakdar, N.; Fabry, B.; Stossel, T. P.; Wang, N.; Weitz, D. A., Filamin A is essential for active cell stiffening but not passive stiffening under external force. *Biophys J* **2009,** 96, (10), 4326-35.
15. Lekka, M.; Laidler, P.; Gil, D.; Lekki, J.; Stachura, Z.; Hrynkiewicz, A. Z., Elasticity of normal and cancerous human bladder cells studied by scanning force microscopy. *Eur Biophys J* **1999,** 28, (4), 312-6.
16. Dey, K.; Roca, E.; Ramorino, G.; Sartore, L., Progress in the mechanical modulation of cell functions in tissue engineering. *Biomater Sci* **2020,** 8, (24), 7033-7081.
17. Navindaran, K.; Kang, J. S.; Moon, K., Techniques for characterizing mechanical properties of soft tissues. *J Mech Behav Biomed Mater* **2023,** 138, 105575.
